# Temperate phage evolve to integrate host stress and quorum signals in lysis–lysogeny decisions

**DOI:** 10.1371/journal.pbio.3003567

**Published:** 2026-01-05

**Authors:** John B. Bruce, Robyn Manley, Elvina Smith, Philippe Carmona, Sylvain Gandon, Edze R. Westra

**Affiliations:** 1 Environment and Sustainability Institute, University of Exeter, Penryn Campus, Exeter, United Kingdom; 2 Strathclyde Institute of Pharmacy and Biomedical Sciences, University of Strathclyde, Glasgow, United Kingdom; 3 Laboratoire de Mathématiques Jean Leray, Université de Nantes, Nantes, France; 4 CEFE, CNRS, Univ Montpellier, EPHE, IRD, Montpellier, France; Monash University, AUSTRALIA

## Abstract

Temperate phage can transmit both horizontally (lytic cycle) and vertically (lysogenic cycle). Many temperate phage have the ability to modify their lysis/lysogeny decisions based on various environmental cues. For instance, many prophage are known to reactivate when SOS stress responses of their host are triggered. Temperate phage infecting *Bacilli* can also use peptide signals (“arbitrium”) to control their lysis/lysogeny decisions. However, information from the arbitrium and SOS systems can be potentially conflicting, and it is unclear how phage integrate information carried by these two different signals when making lysis–lysogeny decisions. Here, we use evolutionary epidemiology theory to explore how phage could evolve to use both systems to modulate lysis/lysogeny decisions in a fluctuating environment. Our model predicts that it can be adaptive for phage to respond to both host SOS systems and arbitrium signaling, as they provide complementary information on the quality of the infected host and the availability of alternative hosts. Using the phage phi3T and its host *Bacillus subtilis*, we show that during lytic infection and as prophage, lysis–lysogeny decisions rely on the integration of information on host condition and arbitrium signal concentrations. For example, free-phage are more likely to lysogenise a stressed host, and prophage are less likely to abandon a stressed host, when high arbitrium concentrations suggest susceptible hosts are unavailable. These experimental results are consistent with our theoretical predictions and demonstrate that phage can evolve plastic life-history strategies to adjust their infection dynamics to account for both the within-host environment (host quality) and the external environment that exists outside of their host (availability of susceptible hosts in the population). More generally, our work yields a new theoretical framework to study the evolution of viral plasticity under the influence of multiple environmental cues.

## Abstract 2

Temperate phage face conflicting signals from host stress and quorum sensing when deciding between lysis and lysogeny. This study shows that bacteriophage integrate information on host condition via SOS responses and on host availability via arbitrium signalling to optimise infection strategies across changing environments.

## 1. Introduction

The lysis/lysogeny decisions of temperate phage have major implications for the spread of the virus and for the dynamics of its bacterial host. The evolution of the virus lysis/lysogeny decision is a classical biological puzzle which has attracted different theoretical approaches to elucidate the within-host processes leading to the viral decision at the scale of the infected bacteria [1,2–4], and the between-host selection acting on this viral trait at the scale of a population of infected bacteria [1,4–6]. Analytical models have recently been developed to demonstrate that the evolutionary stable transmission mode of a temperate phage depends on both the viability of its current host and the availability of susceptible bacteria in the population [7–10]. We developed a theoretical framework to study the evolution of the lysis/lysogeny decision in a fluctuating environment and used this framework to study both the evolution of *fixed* viral strategies (i.e., where the lysogenisation and lysis rates of the virus are not modified by environmental signals) or *plastic* viral strategies (i.e., where the lysogenisation and lysis rates of the virus can be modified by environmental signals) [11,12]. In our previous studies we focused on the evolution of plastic viral strategies where the virus reacts to a single environmental cue (i.e., host stress [12] or arbitrium concentration [11]). Viruses are likely exposed to a multitude of environmental signals in natural conditions which they can sense and respond to, yet theoretical frameworks to analyze the evolution of viral plasticity in these more complex scenarios are currently lacking.

In the following, we expand our theoretical framework to explore how temperate phage should evolve plastic strategies to react to variation in (i) the host physiological state (i.e., the SOS response) and (ii) the availability of susceptible hosts (i.e., arbitrium signals). We predict how viral plasticity should evolve in a range of ecological scenarios with or without fluctuations of the environment, and with or without exposure to stresses that activate the host SOS response. Next, we use the temperate phage phi3T and its host *Bacillus subtilis* to experimentally measure viral plasticity against different cues. These experiments confirm that the lysis–lysogeny decisions of the phage rely on the integration of host stress information and arbitrium concentrations, both during the primary infection of the host, and during the lysogenic phase of the infection (as a prophage).

## 2. Theory: Evolutionary epidemiology in a fluctuating environment

**Fig 1:**
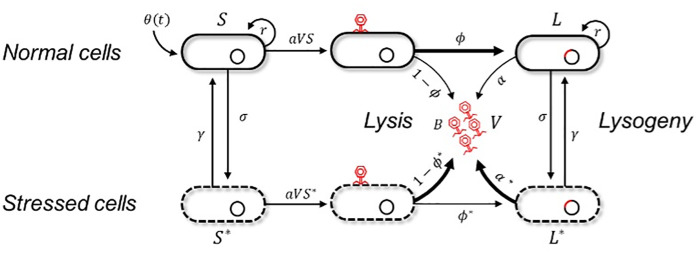
Schematic illustration of the model. This model tracks the densities of susceptible cells (normal S(tand stressed $S^{*}(t)$), lysogenic cells (normal L(tand stressed $L^{*}(t)$) and the density of phage virions V(t). All the parameters of the model are defined in **Table 1** and the whole life cycle is detailed in the Methods section. Note that the traits α, $\alpha^{*}$, ϕ, and $\phi^{*}$ may also vary with the concentration of arbitrium A(t) which is not presented in this figure.

We built an epidemiological model of a well-mixed microbial population consisting of susceptible bacterial cells, bacterial cells harboring a temperate prophage, and free phage particles (Fig 1). The dynamics of the bacterial population is driven by θ(t), the influx of susceptible cells into the population, and we assume that this influx can either be constant or vary periodically with time. We assume a proportion of bacterial cells in the population are stressed—a state in which they have activated the SOS stress response, cannot reproduce, and are more likely to die, but from which they may recover and reproduce successfully. We allow the lysogenisation rates of viral particles and lysis rates of prophage (reactivation) to vary in response to changes in the environment perceived by the phage. We assume that the temperate phage has two distinct types of “sensors”. The first, is the ability to perceive the physiological state of the cell it infects (stressed or unstressed). The second, is the ability to track the concentration of a peptide signal produced by the phage (arbitrium). We assume that the arbitrium signal is produced both by prophage and during infection of susceptible cells by free phage. The information accessible via these two sensors is very different because the first gives access to information on the quality of the currently infected host, while the second gives access to information on the availability of future hosts.

**Table 1:** Main parameters and units of the model. The final column indicates the default parameter values used in our simulations (Figs 2 and S1–S4).

Parameter (units)	Definition	Default value
$\theta (cells.{ml}^{-\text{1}}.h^{-\text{1}})$	Influx of susceptible host cells	$\theta (t)=\theta_{max}1_{\left[\frac{t}{T}<g\right]}$ with $\theta_{max}=\text{2}\text{5}\text{0}$ and g=0.2 θ(t) varies between 0 and 250 and ⟨θ⟩=50
$r(h^{-\text{1}})$	Replication rate of normal cells	1
κ $\left(ml.{cells}^{-\text{1}}\right)$	Density dependent coefficient	${\text{1}\text{0}}^{-\text{3}}$
a $\left(ml.{virus}^{-\text{1}}.h^{-\text{1}}\right)$	Adsorption rate	${\text{1}\text{0}}^{-\text{4}}$
σ	Intensity of stress	Variable
γ $\left(h^{-\text{1}}\right)$	Recovery rate from the stressed state	${\text{1}\text{0}}^{-\text{1}}$
$d_{S}$ $\left(h^{-\text{1}}\right)$	Death rate of normal susceptible cells	${\text{1}\text{0}}^{-\text{1}}$
$d_{L}$ $\left(h^{-\text{1}}\right)$	Death rate of normal lysogenic cells	${\text{1}\text{0}}^{-\text{1}}$
$d_{S^{*}}$ $\left(h^{-\text{1}}\right)$	Death rate of stressed susceptible cells	${\text{1}\text{0}}^{-\text{1}}$
$d_{L^{*}}$ $\left(h^{-\text{1}}\right)$	Death rate of stressed lysogenic cells	${\text{1}\text{0}}^{-\text{1}}$
B $\left(virus.{cell}^{-\text{1}}\right)$	Burst size (number of virions produced after lysis)	10
$d_{V}$ $\left(h^{-\text{1}}\right)$	Death rate of viral particles	${\text{1}\text{0}}^{-\text{1}}$
$\pi_{V}$ $\left(h^{-\text{1}}\right)$	Production rate of arbitrium during lysis	1
$\pi_{L}$ $\left(h^{-\text{1}}\right)$	Production rate of arbitrium during lysogeny	1
$d_{A}$ $\left(h^{-\text{1}}\right)$	Degradation rate of arbitrium	0
δ $\left(h^{-\text{1}}\right)$	Degradation rate of arbitrium by bacteria	10
k	Slope of the shape of the smoothed Heaviside function	10
ϕ	Probability of lysogenisation in normal cells	Variable
α $\left(h^{-\text{1}}\right)$	Reactivation rate of normal cells	Variable
$\phi^{*}$	Probability of lysogenisation in stressed cells	Variable
$\alpha^{*}$ $\left(h^{-\text{1}}\right)$	Reactivation rate of stressed cells	Variable
S $\left(cells.{ml}^{-\text{1}}\right)$	Density of susceptible normal cells	Variable
L $\left(cells.{ml}^{-\text{1}}\right)$	Density of lysogenic normal cells	Variable
$S^{*}$ $\left(cells.{ml}^{-\text{1}}\right)$	Density of susceptible stressed cells	Variable
$L^{*}$ $\left(cells.{ml}^{-\text{1}}\right)$	Density of lysogenic stressed cells	Variable
$N=S+L+S^{*}+L^{*}$ $\left(cells.{ml}^{-\text{1}}\right)$	Total density of cells	Variable
V $\left(virus.{ml}^{-\text{1}}\right)$	Density of viral particles	Variable
A $\left(mol.{ml}^{-\text{1}}\right)$	Concentration of arbitrium	Variable

This model allowed us to assess and predict how the temperate phage should evolve to respond optimally to multiple signals under various environmental scenarios (S1–S4 Figs). Specifically, we obtained the selection coefficient of a phage mutant with altered lysogenisation and lysis rates. To account for multiple signals, we assume that the response to arbitrium signal can evolve independently in normal and in stressed cells. This allows the phage to respond both to changes in host physiological state (normal or stressed) and to fluctuations in susceptible host densities (arbitrium provides indirect information on the availability of susceptible hosts in a fluctuating environment).

Our evolutionary analysis shows that the direction of selection for both traits (lysogeny and lysis) may vary with time and is dictated by the difference between the reproductive value of a prophage relative to the reproductive value of *B* virions (where *B* is the burst size of the virus). Indeed, selection is driven by the contribution of a prophage to the future of the viral population relative to the contribution of a phage that decides to lyse the host and to release *B* new virions. Reproductive values quantify the relative contributions of the different stages of the virus life cycle to the future of the virus population. It thus provides the proper measure to quantify the selection acting on the traits that govern the transitions among these different stages [11,12]. Since the prophage may either infect a stressed or an unstressed cell, we need to keep track of three types of reproductive values to understand the evolution of the lysis/lysogeny decision: (1) the reproductive value $v_{L}$of a prophage in an unstressed cell, (2) the reproductive value $v_{L^{*}}$ of a prophage in stressed cell, (3) the reproductive value $v_{V}$ of a virion. Our model shows that the reproductive value of a prophage in a stressed host is lower than that of an unstressed host (when the virus is unable to respond to the stress level of the host), as these hosts cannot reproduce ($v_{L}>v_{L^{*}})$. Therefore, we expect in a *stable* environment with a constant influx of susceptible cells, that reactivation of prophage or lysis of newly infected hosts will be more favorable in stressed hosts relative to unstressed hosts. In this stable environment the concentration of arbitrium will reach an equilibrium level set by the balance between the production and the degradation of the signal. But the signal does not carry any information that is useful for the phage lysis/lysogeny decision, which therefore should be only based on a host’s physiological state which gives accurate information on the stress level. The joint evolution of the lysogenisation rate and the reactivation rate is expected to yield a coevolutionary stable state where the phage reacts to host stress by a shift towards a lower rate of lysogenisation and higher rate of reactivation (see S1–S4 Figs for detailed analysis of evolutionary stable states in constant versus fluctuating environments, with or without stress).

**Fig 2:**
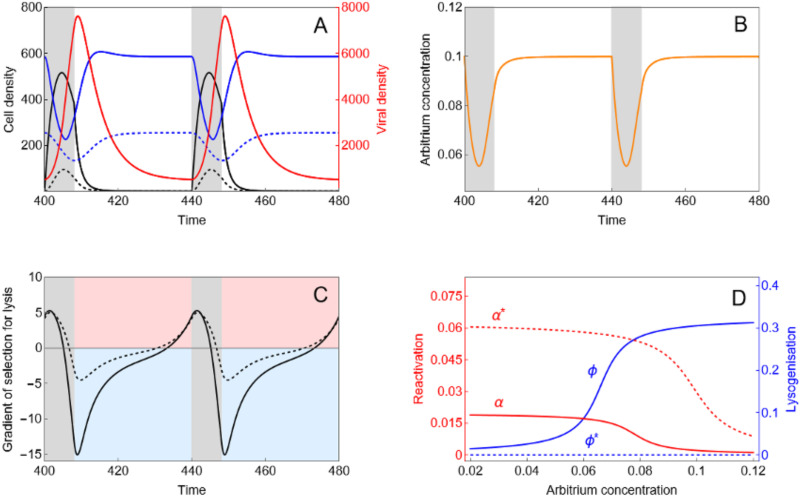
Epidemiological fluctuations drive the evolution viral plasticity to stress and arbitrium concentration. **A)** Fluctuations of the density of the free virus (red) and different cell types (black: susceptible cells, blue: lysogen) where the full lines refer to non-stressed cells and dashed lines refer to stressed cells. The gray shading indicates the time when there is an influx of new uninfected cells in the host population. **B)** Fluctuations in arbitrium signal concentration (orange). **C)** Fluctuations of the sign of the gradient of selection in normal (full line: $Bv_{V}(t)-v_{L}(t)$) and stressed cells (dashed black line: $Bv_{V}(t)-v_{L^{*}}(t)$). The red shading indicates that lysis is favored ($Bv_{V}(t)>v_{L}(t))$, while the blue shading indicates lysogeny is favored ($Bv_{V}(t)<v_{L}(t))$. **D)** evolutionary stable strategies α(A) and ϕ(A) for normal cells and the evolutionary stable strategies $\alpha^{*}(A)$ and $\phi^{*}(A)$ for stressed cells. The model and the parameter values used for the figures are detailed in Table 1 and in the Methods section.

In a fluctuating environment with periodic influx of susceptible cells (Fig 2A), both arbitrium concentrations and the reproductive values of the phage fluctuate with time (Fig 2B and 2C). Selection for lysis is maximized just before the peak in the density of susceptible cells. The phage does not have direct access to the relative densities of hosts, but the ability to track the fluctuations of arbitrium concentration (Fig 2B) may provide an indirect way to obtain this information because selection for lysis coincides with a relatively low concentration of arbitrium (compare Fig 2B and 2C). We show in S5–S7 Figs that this dynamical pattern is robust to variations of several key parameters of the model. Hence, in such a fluctuating environment, we expect that the phage may switch between different lysis/lysogeny strategies when the arbitrium signal reaches a critical level [11]. When the bacteria can be in different physiological states, we can extend the previous analysis to study the joint evolution of phage life-history traits in both stressed and unstressed cells. More specifically, we studied the coevolution between multiple traits of the phage in a fluctuating environment, and we determined the evolutionary stable strategies of the virus in both stressed and unstressed cells (Fig 2D). This analysis shows that in environments with multiple environmental perturbations (i.e., the physiological state of the bacteria and the availability of susceptible hosts) it is adaptive for the phage to use both host SOS and arbitrium signals to make informed decisions on their mode of transmission. Phage infecting a stressed host should always avoid lysogeny, while a phage infecting a normal host should have low rates of rates of lysogeny when low signal concentrations suggest alternative hosts are available, but the rate of lysogenisation should increase with signal concentration (Fig 2D). In the prophage state, the reactivation rate should be higher in stressed cells, but rates of reactivation should drop in both normal and stressed cells (albeit at different rates) when high levels of signal suggest finding another uninfected host is highly unlikely (Fig 2D).

## 3. Experiments: Integration of SOS and arbitrium signals in the lysis–lysogeny decision of a temperate phage

Next, we aimed to experimentally test the qualitative predictions generated by our theoretical model using the previously described model system of *Bacillus subtilis* strain 168 and its arbitrium-producing phage phi3T [11,13–21]. Phi3T is likely to be exposed to stress and to fluctuations in the availability of susceptible cells in its natural environment. Consequently, we expect from the above evolutionary analysis that phage phi3T has evolved plastic strategies to host stress and to arbitrium concentration (Fig 2D).

As *B. subtilis* 168 contains other mobile genetic elements (MGEs) such as the SPbeta prophage and the defective prophages PBSX and skin, we first confirmed that these MGEs do not influence phi3T induction rates [22–24] (S8 Fig). Crucially, although SPbeta was induced in response to phi3T infection, we found no evidence of recombination between SPbeta and phi3T (S9–S11 Figs) and were able to selectively track the replication of phi3T by spot assay on WT *B. subtilis* 168 lawns, which do not support plaque formation by SPbeta (S12 Fig).

**Fig 3:**
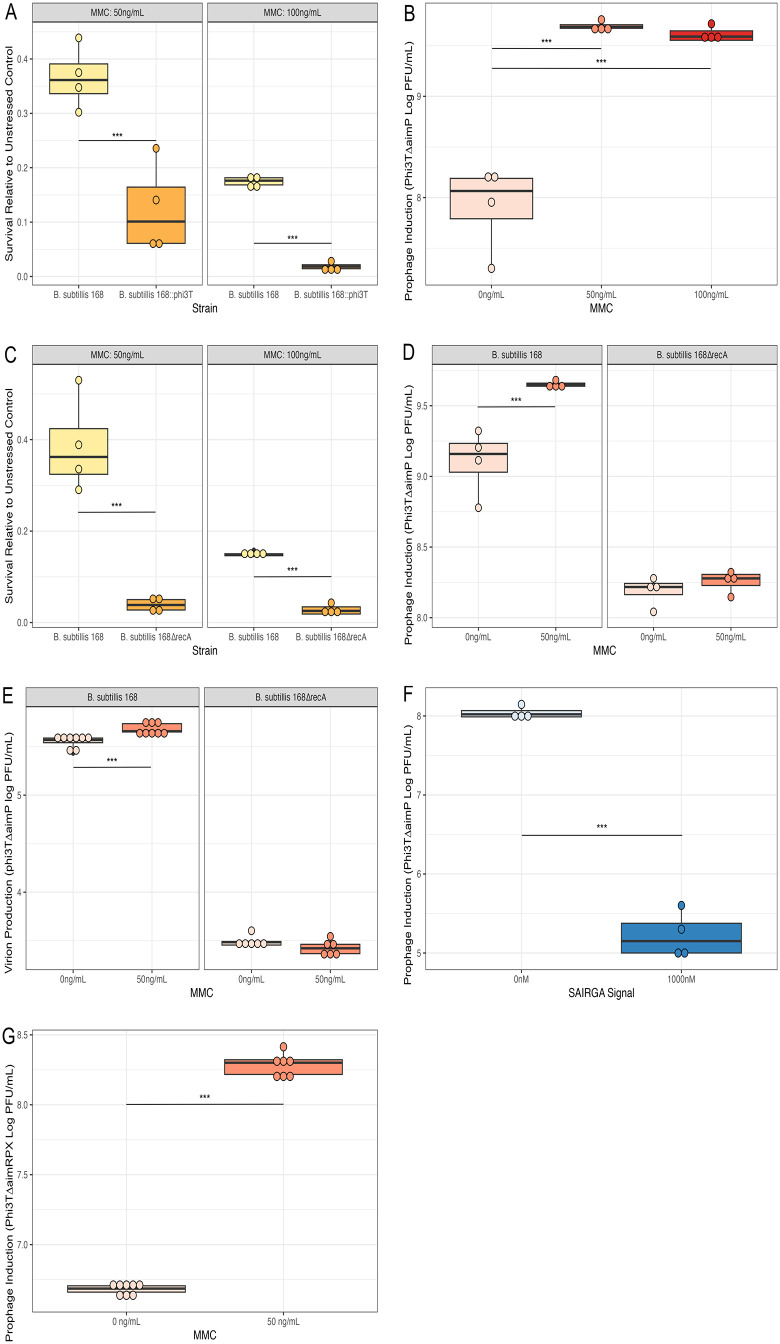
Phi3T prophage sense and respond to both host stress (mitomycin C: MMC) and arbitrium signaling (SAIRGA signal peptide): Box plots show the median and interquartile range, with individual data points plotted. Plaque-forming units (PFU/mL) and colony-forming units (CFU/mL) are presented on the log_10_ scale. Tukey-adjusted *p* < 0.05 comparisons are marked with * *p* < 0.05, ** *p* < 0.01, *** *p* < 0.001. Colors indicate comparison type: MMC concentrations (shades of red, darker with higher concentrations), Arbitrium signaling peptide concentrations (shades of blue, darker with higher concentrations), and strain comparisons (shades of yellow for each strain). **A)** Survival of *B. subtilis* 168 and *B. subtilis* 168 carrying a phi3TΔ*aimP* prophage (*B. subtilis* 168:: phi3TΔ*aimP*) when exposed to stress (50 or 100 ng/mL of MMC) relative to unstressed (MMC 0 ng/mL) control (*N* = 4 replicates per strain and MMC treatment). **B)** Prophage induction of phi3TΔ*aimP* (Log PFU/mL) in *B. subtilis* 168 host exposed to 0, 50, and 100 ng/mL of MMC (*N* = 4 replicates per MMC treatment). **C)** Survival of cells with a functional SOS system (WT: *B. subtilis* 168), and without a functional SOS system (*B. subtilis* 168Δ*recA*) when exposed to stress (50 or 100 ng/mL of MMC) relative to unstressed (MMC 0 ng/mL) control (*N* = 4 replicates per strain and MMC treatment). **D)** Prophage induction of phi3TΔ*aimP* (Log PFU/mL) in WT host (*B. subtilis* 168) and host without a functional SOS system (*B. subtilis* 168Δ*recA*), in the presence (MMC 50 ng/mL) or absence (MMC 0 ng/mL) of stress (*N* = 4 replicates per MMC treatment). **E)** Virion production from a single round of infection at MOI of 0.1 in WT host (*B. subtilis* 168) and hosts without a functional SOS system (*B. subtilis* 168Δ*recA*), in the presence (MMC 50 ng/mL) or absence (MMC 0 ng/mL) of stress (*N* = 4 replicates per MMC treatment). **F)** Prophage induction of phi3TΔ*aimP* (Log PFU/mL) in host *B. subtilis* 168 host without a functional SOS system (*B. subtilis* 168Δ*recA*) in the presence (1,000 nM) or absence (0 nM) of arbitrium signaling peptide (SAIRGA) (*N* = 4 replicates per SAIRGA treatment). **G)** Prophage induction of a phage mutant lacking a functional arbitrium signaling system (phi3T∆*aimRPX*) in host (*B. subtilis* 168) in the presence (MMC 50 ng/mL) or absence (MMC 0 ng/mL) of stress (*N* = 8 replicates per MMC treatment). The data underlying Fig 3A–3G is available in S1 Data.

Having validated that the WT *B. subtilis* 168 is an appropriate model host to study phi3T lysis/lysogeny decisions, we next wanted to examine whether this phage adjusts its lysis/lysogeny decision when the host SOS response is activated. We therefore compared survival of a prophage-free host (*B. subtilis* 168) and a phi3T lysogen (*B. subtilis* 168::phi3T) upon exposure to stress in the form of a DNA-damaging agent, mitomycin C (MMC). Consistent with previous studies that suggest that phi3T induces lytic replication in response to the host SOS response [16,20], we found that hosts harboring phi3T prophage were significantly less likely to survive compared to uninfected cells (*F*1,14 = 53.362, *P* < 0.001), when exposed to both low (Tukey-adjusted *p*-value 50 ng/mL: *P* < 0.001) and high (100 ng/mL: *P* < 0.001) concentrations of MMC (Fig 3A). This suggests that phi3T prophage are capable of sensing stress experienced by the host and respond by reactivating and lysing the host, subsequently decreasing host survival. To further corroborate the idea that prophage increase induction in response to stress, we next quantified virion production in the presence and absence of MMC. This revealed that virion production through phi3TΔaimP prophage induction in the WT *B. subtilis* 168 host differed significantly dependent on MMC exposure (*F*2,11 = 88.469, *P* < 0.001), with increased virion production at low and high concentrations of MMC when compared to the unstressed control (0 ng/mL Tukey-adjusted *p*-value: 50 ng/mL *P* < 0.001 and 100 ng/mL *P* < 0.001) (Fig 3B). These results confirm that phi3T prophage sense when their current host is at risk of becoming inviable and respond by switching to horizontal (lytic) transmission.

Many phage sense host stress through direct interactions between components of the host SOS response pathway and regulators of the lysis/lysogeny switch [25–29]. To examine whether this is also the case for phage phi3T, we first compared (as a control) the survival *of B. subtilis* hosts with (wild-type (WT) *B. subtilis* 168) and without a functional SOS system (*B. subtilis* 168Δ*recA*) in the absence of phage infection. We found that hosts with a functional SOS system were significantly more likely to survive MMC exposure (*F*1,14 = 33.815, *P* < 0.001) at both low (Tukey-adjusted *p*-value 50 ng/mL: *P* < 0.001) and high concentrations (100 ng/mL: *P* < 0.001) (Fig 3C). These results reiterate previous studies demonstrating that *B. subtilis* can remain viable after exposure to a DNA damaging agent, and that the SOS system is central to sensing and responding to these stresses [30]. Next, we tested our hypothesis that prophage induction is higher in WT hosts (*B. subtilis* 168) than in hosts without a functional SOS system (*B. subtilis* 168Δ*recA*). We found that prophage induction increased significantly in response to stress, but only in the WT *B. subtilis* 168 host (interaction between MMC level and host type on prophage induction: *F*1,13 = 81.253, *P* < 0.001; Tukey-adjusted *p*-value MMC 0 –50 ng/mL: *P* < 0.001). Induction was lower in hosts lacking a functional SOS system, likely due to slower growth rates impacting phage replication and loss of background SOS activation, and prophage induction did not significantly increase in response to MMC exposure (*B. subtilis* 168Δ*recA* MMC 0–50 ng/mL: *P* = 0.999) (Fig 3D). To test whether phi3T also senses and responds to stress when making lysis–lysogeny decisions upon infection of a new host, we compared virion production from a single round of infection at an MOI of 0.1 in WT hosts (*B. subtilis* 168) and hosts without a functional SOS system (*B. subtilis* 168Δ*recA*). When infecting new hosts, virion production increased significantly in response to stress, but only in the WT host (interaction between MMC level and host type on virion production: *F*1,25 = 14.172, *P* = 0.001; *B. subtilis* 168 Tukey-adjusted *p*-value MMC 0–50 ng/mL: *P* < 0.001). Conversely, virion production did not increase in mutant hosts lacking a functional SOS system (*B. subtilis* 168Δ*recA* MMC 0–50 ng/mL: *P* = 1.000) (Fig 3E). These results demonstrate that phi3T uses the hosts SOS system to gather information on its physiological state and responds to stress experienced by the host as both a prophage and when making lysis–lysogeny decisions during infection.

To explore whether SOS responses are necessary for responding to arbitrium signals in phi3T, we measured prophage induction in *B. subtilis* 168Δ*recA* strains with and without arbitrium signal. We found that prophage responded to arbitrium signaling peptide (SAIRGA) in the absence of information on host stress (*F*1,7 = 103.33, *P* < 0.001), significantly reducing prophage induction (0 nM Tukey-adjusted *p*-value: 1,000 ng/mL *P* < 0.001) (Fig 3F). To determine whether phage can respond to stress independently of arbitrium signaling, we measured prophage induction from WT host (*B. subtilis* 168) in the presence and absence of stress using a phage mutant lacking a functional arbitrium signaling system (phi3T∆*aimRPX*). We found that prophage induction in phi3T∆*aimRPX* increased significantly in response to stress (*F*1,14 = 203.19, *P* < 0.001; MMC 0–50 ng/mL: *P* < 0.001) (Fig 3G). This result confirms that host stress is not essential for prophage induction, and that phi3T can independently sense and respond to host stress and arbitrium signals.

**Fig 4:**
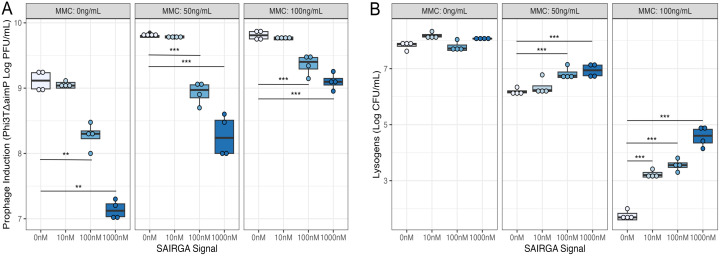
Signaling peptides (SAIRGA signal) moderate phi3T prophage induction under stress (mitomycin C: MMC). Box plots show the median and interquartile range, with individual data points plotted. Plaque-forming units (PFU/mL) and colony-forming units (CFU/mL) are presented on the log_10_ scale. Tukey-adjusted *p* < 0.05 comparisons are marked with * *p* < 0.05, ** *p* < 0.01, *** *p* < 0.001. Colors indicate increasing concentrations of arbitrium signaling peptide (SAIRGA): progressively darker shades of blue represent higher concentrations. **A)** Prophage induction of phi3TΔ*aimP* (Log PFU/mL) in *B. subtilis* 168 and **B)** host survival (Lysogens: CFU/mL), after exposure to different levels of stress (MMC: 0, 50, and 100 ng/mL) and arbitrium signaling peptides (SAIRGA signal: 0, 10, 100, and 1,000 nM) (*N* = 4 replicates per MMC and SAIRGA treatment). The data underlying Fig 4A and 4B is available in S2 Data.

While this demonstrates that phi3T can respond to both the presence of signaling peptides and host physiological state, it remains unclear whether phi3T integrates these two pieces of information to make more informed decisions, as predicted by our model. If the two sources of information are integrated, we might expect that prophage response to stress will be markedly different when arbitrium signals are present versus when they are absent (Fig 2D). To explore this, we compared both prophage induction and host survival in response to different combinations of host stress and signaling peptides. We cultured phi3t∆*aimP* lysogens in LB supplemented with different combinations of both synthetic signal (SAIRGA) and MMC and quantified prophage induction. We found that increasing concentrations of arbitrium signaling peptide significantly decreased virion production (*F*2,45 = 44.557, *P* < 0.001), in absence of stress (MMC 0 ng/mL; SAIRGA signal 0 nM Tukey-adjusted *p*-value: 10 nM *P* = 0.9122, 100 nM *P* = 0.0096, 1,000 nM *P* = 0.0022), low levels of stress (MMC 50 ng/mL; SAIRGA signal 0 nM Tukey-adjusted *p*-value: 10 nM *P* = 0.9122, 100 nM *P* < 0.001, 1,000 nM *P* < 0.001), and high levels of stress (MMC 100 ng/mL; SAIRGA signal 0 nM Tukey-adjusted *p*-value: 10 nM *P* = 0.1905, 100 nM *P* < 0.001, 1 000 nM *P* < 0.001) (Fig 4A). Increasing levels of signaling peptide had different effects on host survival (lysogen CFU/mL) dependent on stress level (interaction between MMC level and signal concentration on lysogen CFU/mL: *F*2,42 = 9.6642, *P* < 0.001). In the absence of stress, increasing levels of signaling peptide had no significant effect on host survival (MMC 0 ng/mL; SAIRGA signal 0 nM Tukey-adjusted *p*-value: 10 nM *P* = 0.0860, 100 nM *P* = 0.9854, 1,000 nM *P* = 0.339). Conversely, when exposed to stress (MMC), increasing amounts of signaling peptide significantly increased host survival at low (MMC 50 ng/mL; SAIRGA signal 0 nM Tukey-adjusted *p*-value: 10 nM *P* = 0.6884, 100 nM *P* < 0.001, 1,000 nM *P* < 0.001) and high (MMC 100 ng/mL; SAIRGA signal 0 nM Tukey-adjusted *p*-value: 10 nM *P* < 0.001, 100 nM *P* < 0.001, 1,000 nM *P* < 0.001) levels of stress (Fig 4B). This shows that the prophage response to stress is suppressed when high arbitrium signal concentrations indicate that alternative susceptible hosts are unavailable.

**Fig 5:**
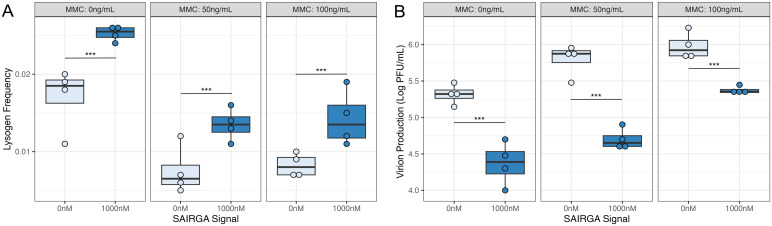
Signaling peptides (SAIRGA signal) moderate phi3T lysis–lysogeny decisions under stress (mitomycin C: MMC). Box plots show the median and interquartile range, with individual data points plotted. Plaque-forming units (PFU/mL) are presented on the log_10_ scale. Tukey-adjusted *p* < 0.05 comparisons are marked with * *p* < 0.05, ** *p* < 0.01, *** *p* < 0.001. Colors indicate concentrations of arbitrium signaling peptide (SAIRGA). **A)** Frequency of phi3T∆*aimP*::spc lysogens within a population of *B. subtilis* 168 and **B)** and virion production of phi3T∆*aimP*::spc (Log PFU/mL) resulting from lytic infection of host at an MOI of 0.1 after exposure to different levels of stress (MMC: 0, 50, and 100 ng/mL) in the presence (SAIRGA signal: 1,000 nM) or absence (SAIRGA signal: 0 nM) of arbitrium signaling peptides (*N* = 4 replicates per MMC and SAIRGA treatment). The data underlying Fig 5A and 5B is available in S3 Data.

Our model predicts that free phage should also integrate information on the host physiological state (stress) and the availability of uninfected bacteria (arbitrium) when making lysis–lysogeny decisions following the infection of a new host (i.e., in the transition from lytic to lysogenic replication) (Fig 2D). To explore this, we compared lysogen formation and virion production in response to infections with free phage particles at an MOI of 0.1 while varying concentrations of MMC and signaling peptides. We found that lysogen formation, measured after a single round of infection, was significantly greater when signal was present (*F*2,21 = 37.194, *P* < 0.001), in absence of stress and low and high levels of stress (SAIRGA signal 0 nM compared to 1,000 nM Tukey-adjusted *p*-value: *P* < 0.001 at MMC 0, 50, and 100 ng/mL) (Fig 5A). We also found that the presence of signaling peptide significantly reduced virion production by phage phi3T∆*aimP::spc* (*F*2,21 = 106.86, *P* < 0.001), in absence of stress and low and high levels of stress (SAIRGA signal 0 nM compared to 1,000 nM Tukey-adjusted *p*-value: *P* < 0.001 at MMC 0, 50, and 100 ng/mL) (Fig 5B). While we cannot distinguish between failure to form lysogens and lysogens forming then reactivating, these results indicate that free phage are more likely to lysogenise in the absence of stress and when arbitrium signal concentrations suggest alternative susceptible hosts are unavailable.

## 4. Discussion

We have shown, using novel theory and experiments, that phi3T phage integrate information from arbitrium signaling and from host stress responses when making both prophage induction and lysis/lysogeny decisions. Both horizontal and vertical transmission are inherently risky: a host may become unviable, preventing vertical transmission as a prophage, while virions produced during lysis risk failing to find an alternative host. Ultimately, the optimal mode of transmission of a temperate phage is contingent on both the viability of its current host and the availability of new susceptible hosts in the local population. Integrating information on the within-host and external environment allows phage to make more informed decisions: staying in a stressed but potentially viable host when finding a susceptible alternative is unlikely, or abandoning a moribund host when low signal concentrations suggest susceptible hosts may be available.

The present study combines a theoretical approach which predicts the evolution of virus phenotypes under different ecological scenarios, with experimental measures of these phenotypes after exposing the bacteria and the phage to different levels of stress and arbitrium. This combination of approaches yields important insights on the evolutionary forces driving viral plasticity. Yet, a direct quantitative test of the theory with experiments is currently out of reach. First, many steps of the life cycle remain difficult to characterize (e.g., the production and the persistence of arbitrium signals) which means that several parameters of the model are difficult to estimate precisely. Even if the dynamical patterns driving the evolution of viral plasticity (i.e., the negative covariance between the selection for lysis and the concentration of arbitrium) are robust to a variation of some parameter values (Figs S5–S7) a more extensive exploration of the sensitivity analysis of our theoretical predictions remains to be carried out. This is particularly important to explore the validity of the quantitative predictions of our model. Second, our theoretical model makes predictions on the evolution of phenotypic traits (e.g., probability of lysogenisation, rate of reactivation) while our bulk experiments allow us to obtain measures of densities of lysogens (colony-forming units, CFU) and densities of free virus (plaque-forming units, PFU). The development of single-cell approaches may soon provide new ways to measure viral phenotypes at the scale of the infected cell [31–33]. This single-cell monitoring will help to bridge the gap between the theory and the biology of viral plasticity by estimating parameter values with high confidence and by monitoring and accounting for heterogeneity in signaling and phenotypic outcomes within the population. Third, our comparison between theory and experiments is based on the hypothesis that the virus we study has evolved to a temporally fluctuating environment, but we do not test directly the influence of these fluctuations on the evolution of the virus. For instance, we show in the Methods section that plasticity to arbitrium concentration should not evolve in a stable environment. This prediction remains to be tested with evolution experiments under different regimes of temporal fluctuation. Our work provides a first step towards a validation of the theoretical predictions of our evolutionary model.

That said, our work provides key insights and a new theoretical framework to understand the evolution of multiple sensors in phage genomes and the existence of molecular interactions between these sensors. For example, while the molecular mechanisms of signal SOS and arbitrium signal integration are yet to be determined for phage phi3T, recent studies suggest potential mechanisms for interactions between SOS and arbitrium signaling in related phage. Specifically, in the SPbeta phage arbitrium and SOS signals may be integrated downstream of *aimX* expression, whereas these signals may jointly control the expression of *aimX* in the φ106 phage [20]. It is possible that mechanistic variations in the way these systems interact alters how information is integrated, potentially tipping the balance in favor of one signal over another. Future work will help to clarify if and how the benefits of different mechanisms to integrate multiple signals depends on environmental variables.

While here we have focused on host SOS systems and phage arbitrium signaling, host and population-level information can be gleaned from other sources. For example, a mobile conjugative element infecting *Bacilli* uses molecular signaling to avoid excision when surrounded by cells also carrying the signaling system, but appears to use information from a host transcriptional state regulator, AbrB, to ensure it only attempts excision and transfer when cells densities are high in stationary phase [34]. Recent work has also shown that Vibriophage often carry orphan quorum-sensing receptors, potentially allowing the phage to eavesdrop on their hosts communication systems, to obtain information on local population densities, and time their reactivation to periods of high host densities [35]. Bacteria have an incredible array of mechanisms for sensing biotic and abiotic conditions [36–38] and information from these sensing systems allows bacteria to sense and adapt to their surroundings—future work is likely to find further examples of bacteriophage appropriating these host sensing systems to optimize their own reproductive decisions.

The new theoretical framework we have developed allows us to predict the evolution of the lysis–lysogeny decision in a scenario where the virus integrates two sources of information. However, some bacteriophage may integrate information from additional sources, both from the host and externally, to make more informed lysis–lysogeny decisions. Utilizing multiple sources of information may also be important for competition with other phage [39]. It is becoming apparent that polylysogeny is widespread and that prophage compete for host resources upon induction [40]. For example, in lamboid phage infecting *E. coli* induction of double-lysogens consistently resulted in one phage increasing in frequency relative to its competitor [41]. While DNA damage is the canonical prophage induction cue, evidence suggests that phage may also sense and respond to other host stressors by inducing and lysing the host [42–43]. The ability to gather information on multiple sources of host stress, and the external environment, may allow phage to get ahead of other lysogens in the race for a stressed-hosts resources [39]. The theoretical framework presented here can readily be extended to explore if and when the integration of multiple other sources of information is evolutionarily beneficial to temperate phage in fluctuating environments.

## 5. Methods

### Resource availability

Further information and requests for resources and reagents should be directed to and will be fulfilled by Edze Westra (E.R.Westra@exeter.ac.uk).

## 6. Theory

### Epidemiological model

We model the epidemiological dynamics of a well-mixed population of bacteria infected by a temperate phage to track the density of susceptible cells (S), lysogenic cells (L), and free virus particles (V). We assume there is an influx θ(t) of susceptible cells in the bacteria population. This influx can either be constant (i.e., $\theta (t)=\theta_{\text{0}}$) or may vary periodically with time (we explore both scenarios below). Both susceptible and lysogenic cells have a per-capita birth rate r(1−κN), where N=S+L is the total density of the bacteria population and κ is a density-dependent coefficient. Susceptible and lysogenic cells have per-capita death rates $d_{S}$ and $d_{L}$, respectively. Free virus particles adsorb to bacterial cells at rate a. Adsobtion to susceptible cells may either result in lysogenisation of the cell with probability ϕ, or, with probability 1−ϕ, to lysis. Upon lysis, B virus particles are produced, and they each have a per-capita death rate $d_{V}$ (see Table 1 for a definition of all parameter values and Fig 1 for a schematic description of the life cycle). As discussed in the main text, many parameters of this model remain unknown. However, an extensive exploration of the dynamics of this model revealed that the results we discuss in this paper are robust across a wide parameter space (S5–S7 Figs). This robustness led us to choose a set of parameter values that maximize the clarity of the argument illustrated in Fig 2. For instance, the periodicity and the magnitude of the fluctuations (i.e., the function θ(t)) we used in Table 1 and Fig 2 was used to illustrate that what drives the evolution of plasticity is the covariation between the concentration A(t) of arbitrium and the density S(t) of susceptible hosts. Then we carried out an exploration of the evolution of the virus under a series of environmental scenarios.

We model stress (e.g., exposure to mitomycin) with the parameter σ which changes the state of cells to a stressed state (indicated with a *). We assume that stressed cells are in a physiological state where they cannot reproduce (and they may suffer from an increased death rate). Yet, stressed cells may eventually recover from the stressed state at a rate γ. This yields the following dynamical equations:


$$\dot{S}=\theta +rS(\text{1}-\kappa N)-\left(aV+d_{S}+\sigma \right)S+\gamma S^{*}$$

$${\dot{S}}^{*}=\sigma S-\left(aV+d_{S^{*}}+\gamma \right)S^{*}$$

$$\dot{L}=rL(\text{1}-\kappa N)+a\phi VS-\left(\alpha +d_{L}+\sigma \right)L+\gamma L^{*}$$

$${\dot{L}}^{*}=\sigma L+a\phi^{*}VS^{*}-\left(\alpha^{*}+d_{L^{*}}+\gamma \right)L^{*}$$

$$\dot{V}=aBV\left((\text{1}-\phi )S+\left(\text{1}-\phi^{*}\right)S^{*}\right)+\left(\alpha L+\alpha^{*}L^{*}\right)B-\left(aN+d_{V}\right)V$$ (1)

For the sake of simplicity, we dropped the dependence to time, but all densities vary with time. Besides, several other parameters of the model may also vary with time. For instance, the influx of susceptible cells or the death rate of bacterial cells may be affected by time-varying fluctuations of some component of the environment. These fluctuations of the environment may also affect the life-history decision of the virus and in particular the lysogenisation and the reactivation rate of the virus. We detail below how to analyze the evolution of these two traits in response to (i) the level of stress of the cell and (ii) the concentration of *arbitrium*, a signaling molecule produced by the phage.

The following equation describes the dynamics of the concentration A of arbitrium:


$$\dot{A}=\pi_{V}aV\left(S+S^{*}\right)+\pi_{L}\left(L+L^{*}\right)-\left(d_{A}+\delta N\right)A$$ (2)

The dynamics of arbitrium results from the production of the peptide at rates $\pi_{V}$ and $\pi_{L}$ during lysis and lysogeny, respectively, and the degradation of arbitrium in the environment (at rate $d_{A}$) and by the bacterial cells (at rate δ).

### Evolutionary model

To understand and predict life-history evolution, we need to determine the fate of viral mutations that affect the lysogenisation and/or the reactivation rate. We thus have to determine the growth rate of a mutant after its appearance in a viral population dominated by a WT genotype. Since the virus may appear in three distinct states, a prophage in non-stressed lysogenic bacteria (L), in a stressed lysogenic bacteria ($L^{*}$) or in a virion outside the cell (V), we can use the following matrix to describe the dynamics of the mutant:


$$M=(\begin{array}{ccc} (\text{1}-\kappa N)-\left(\alpha_{m}(A)+d_{L}+\sigma \right) & \gamma & a\phi_{m}(A)S\\ \sigma & -\left(\alpha_{m}^{*}(A)+d_{L^{*}}+\gamma \right) & a\phi_{m}^{*}(A)S^{*}\\ \alpha_{m}(A)B & \alpha_{m}^{*}(A)B & a\left(\left(\text{1}-\phi_{m}(A)\right)S+\left(\text{1}-\phi_{m}^{*}(A)\right)S^{*}\right)B-\left(aN+d_{V}\right) \end{array})$$ (3)

where the coefficients $M_{ij}$ of the matrix M refer to the production of a mutant virus in state i by a mutant virus in state j. The selection on the trait z at time t is determined by the instantaneous selection gradient:


$$S_{z}(t)=v(t).\frac{\partial M(t)}{\partial z}.f(t)=\sum_{i}\sum_{j}v_{i}(t)\frac{\partial M_{ij}(t)}{\partial z}f_{j}(t)$$ (4)

where $v_{i}(t)$ is the individual reproductive value of a virus in class $i\in \left\{L,L^{*},V\right\}$ and $f_{j}(t)$ is the frequency of the virus in class $j\in \left\{L,L^{*},V\right\}$. In other words, $\frac{\partial M_{ij}(t)}{\partial z}$ measures the influence of a variation of the life-history trait z induced by the mutation on one component of fitness ($M_{ij}$) weighted by the “quantity” of the virus in class j and the “quality” of class i [12]. This instantaneous selection gradient is very useful to describe selection in temporally variable environments and thus to study the evolution of viral plasticity. We need three things to compute this gradient: (i) the frequency of the different classes, (ii) the reproductive value of the virus in the different classes, (iii) the effect of the mutation on each component of the transition matrix 𝐌. We derive these three quantities below.

#### Class frequencies.

We compute the dynamics of class frequencies in the resident system which yields:


$${\dot{f}}_{L}=\left(r(\text{1}-\kappa N)-\left(\alpha +d_{L}+\sigma \right)\right)f_{L}+\gamma f_{L^{*}}+a\phi Sf_{V}-r_{w}f_{L}$$

$${\dot{f}}_{L^{*}}=\sigma f_{L}-\left(\alpha^{*}+d_{L^{*}}+\gamma \right)f_{L^{*}}+a\phi^{*}S^{*}f_{V}-r_{w}f_{L^{*}}$$

$${\dot{f}}_{V}=\alpha Bf_{L}+\alpha^{*}Bf_{L^{*}}+\left(a(\text{1}-\phi )B\left(S+S^{*}\right)-\left(aN+d_{V}\right)\right)f_{V}-r_{w}f_{V}$$ (5)

where $r_{w}$ is the per-capita growth rate of the monomorphic resident population [12].

#### Reproductive values.

Similarly, following Lion and Gandon 2022 [12], we can track the dynamics of reproductive values using:


$${\dot{v}}_{L}=\left(\left(\alpha +d_{L}+\sigma \right)-r(\text{1}-\kappa N)\right)v_{L}-\sigma v_{L^{*}}-\alpha Bv_{V}+r_{w}v_{L}$$

$${\dot{v}}_{L^{*}}=-\gamma v_{L}+\left(\alpha^{*}+d_{L^{*}}+\gamma \right)v_{L^{*}}-\alpha^{*}Bv_{V}+r_{w}v_{L^{*}}$$

$${\dot{v}}_{V}=-ab\phi Sv_{L}-ab\phi^{*}S^{*}v_{L^{*}}-\left(ab(\text{1}-\phi )B\left(S+S^{*}\right)-\left(aN+d_{V}\right)\right)v_{V}+r_{w}v_{V}$$ (6)

#### Evolution of reactivation.

Using (3) and (4), we show that the gradient of selection on reactivation in normal cells is:


$$S_{\alpha }(t)=\left(Bv_{V}(t)-v_{L}(t)\right)f_{L}(t)$$ (7a)

Similarly, we show that the gradient of selection on reactivation in stressed cells is:


$$S_{\alpha^{*}}(t)=\left(Bv_{V}(t)-v_{L^{*}}(t)\right)f_{L^{*}}(t)$$ (7b)

In other words, selection for reactivation may vary with time and the direction of selection is governed by the difference between the reproductive value $v_{L}(t)$ (resp. $v_{L^{*}}(t)$) of a prophage in normal cells (resp. stressed cells) and the reproductive value $v_{V}(t)$ of B virus particles.

#### Evolution of lysogenisation.

The same approach can be used to derive the gradient of selection on lysogenisation in normal cells:


$$S_{\phi }(t)=-\left(Bv_{V}(t)-v_{L}(t)\right)a\hat{S}(t)f_{V}(t)$$ (8a)

And the gradient of selection on lysogenisation in stressed cells:


$$S_{\phi^{*}}(t)=-\left(Bv_{V}(t)-v_{L^{*}}(t)\right)a{\hat{S}}^{*}(t)f_{V}(t)$$ (8b)

Hence, selection on traits affecting stressed cells ($\alpha^{*}$ and $\phi^{*}$) is very similar to selection on traits affecting non-stressed cells (α and ϕ) except that the sign of selection depends on the reproductive value of $L^{*}$ cells instead of L cells. Since $L^{*}$ cannot reproduce we generally expect to have $v_{L}(t)>v_{L^{*}}(t)$ because the prophage can benefit from vertical transmission and we thus expect that reactivation will evolve more readily in stressed cells. Fluctuations in the influx of susceptible cells, however, are going to allow $S_{\alpha }(t)$, $S_{\alpha^{*}}(t)$, $S_{\phi }(t)$ and $S_{\phi^{*}}(t)$ to fluctuate with time. The direction of selection on trait X will depend on the selection gradient averaged over one period of the fluctuation when the resident system sits on its periodic attractor [11]:


$${\hat{S}}_{X}=\left\langle S_{X}(t)\right\rangle =\frac{\text{1}}{T}\int_{t}^{t+T}S_{X}(t)dt$$ (9)

with $X\in \left\{\alpha ,\alpha^{*},\phi ,\phi^{*}\right\}$

#### Coevolution between reactivation and lysogenisation.

Next, we determine the long-term coevolutionary outcome between the different life-history traits of the phage. The above selection gradients, equations (7) and (8), can be used to determine the direction of phenotypic evolution and thus to identify the long-term coevolutionary equilibrium. This equilibrium corresponds to a point in the phenotypic space where ${\hat{S}}_{X}=\text{0}$ with $X\in \left\{\alpha ,\alpha^{*},\phi ,\phi^{*}\right\}$. This coevolutionary equilibrium is expected to depend on the environment and we will discuss different epidemiological scenarios. Before studying the evolution of viral pasticity with arbitrium and with stress, we focus on the evolution of fixed life-history strategies (α and ϕ) that govern the lysis/lysogeny decision.

### Epidemiological scenarios

#### Constant environment.

**Without plasticity to arbitrium and without stress:** As pointed out in a previous study [11], in a constant environment (i.e., $\theta (t)=\theta_{\text{0}}$), and in the absence of stress (i.e., σ=0), the phage evolves towards evolutionary stable lysogenisation and reactivation strategies ($\alpha^{\cdot }$ and $\phi^{\cdot }$). Using equations (7) and (8) we see that the direction of selection is given by the sign of $S_{\alpha }$ and $S_{\phi }$, which is given by the sign of $B{\hat{v}}_{V}-{\hat{v}}_{L}$ and the sign of ${\hat{v}}_{L}-B{\hat{v}}_{V}$, respectively. In a constant environment, we can use equation (6) and in particular ${\dot{v}}_{L}=\text{0}$, to show that the equilibrium reproductive values ${\hat{v}}_{L}$ and ${\hat{v}}_{V}$ satisfy the following condition:


$${\hat{v}}_{L}-B{\hat{v}}_{V}=\left(r(\text{1}-\kappa N)-d_{L}\right)\frac{{\hat{v}}_{L}}{\alpha }$$ (10)

In other words, the sign of ${\hat{v}}_{L}-B{\hat{v}}_{V}$ is given by the sign of $r(\text{1}-\kappa N)-d_{L}$ because $\frac{{\hat{v}}_{L}}{\alpha }>\text{0}$. At equilibrium, we can also use equation (1) and in particular $\dot{S}=\text{0}$ to show that:


$$r(\text{1}-\kappa N)-d_{L}=a\hat{V}-\frac{\theta_{\text{0}}}{\hat{S}}$$ (11)

This means that selection for higher values of α (and lower values of ϕ) occurs as soon as $\theta_{\text{0}}>a\hat{V}\hat{S}$. In other words, it is adaptive to increase the rate of reactivation when $\theta_{\text{0}}$, the influx of new susceptible cells, is higher than $a\hat{V}\hat{S}$, the rate at which susceptible cells become infected. Yet, to find the evolutionary stable lysogenisation and reactivation strategies ($\phi^{\cdot }$ and $\alpha^{\cdot }$) we need to determine how the equilibrium densities $\hat{V}$ and $\hat{S}$ vary with the phenotypes of the virus (ϕ and α). We thus need to solve ${\hat{S}}_{\alpha }={\hat{S}}_{\phi }=\text{0}$ numerically to find the values of $\alpha^{\cdot }$ and $\phi^{\cdot }$. Using the default parameter values presented in Table 1, we find that an infinite number of strategies verify (11) (S1 Fig). Indeed, in a constant environment we can use equations (7) and (8) to show that ${\hat{S}}_{\alpha }$ and ${\hat{S}}_{\phi }$ have opposite signs (i.e., ${\hat{S}}_{\alpha }\propto {\hat{S}}_{\phi }$). The direction of evolution on α and on ϕ is governed by the quantity ${\hat{v}}_{L}-B{\hat{v}}_{V}$ and there is an infinity of pairs of values for $\alpha^{\cdot }$ and $\phi^{\cdot }$ that verify ${\hat{v}}_{L}-B{\hat{v}}_{V}=\text{0}$.

**Without plasticity to arbitrium but with plasticity to stress:** When σ>0 a fraction of the cells are stressed and we can use (6) to compute the reproductive values $v_{L}$ and $v_{L^{*}}$ of normal and stressed prophage. Because we assume stressed cells cannot reproduce, the reproductive value $v_{L}$ is expected to be higher than $v_{L^{*}}$ (in the absence of plasticity). Consequently, when $\left(Bv_{V}-v_{L}\right)=\text{0}$ (i.e., the fixed strategy of the virus is optimal in the normal cells, see equations (7a) and (8a), it implies that $\left(Bv_{V}-v_{L^{*}}\right)>\text{0}$, which selects for plasticity: higher reactivation rates (equation (7b)) and lower lysogenisation rates (equation (8b)) when phage are infecting stressed cells.

#### Fluctuating environment.

**Without plasticity to arbitrium and without stress:** The above scenarios are not very realistic because bacteria live in temporally variable environments. Next, to account for this temporal variability, we assume that the influx of susceptible cells fluctuates periodically as a square wave with period T=40, between θ=0 for 80% of the period (i.e., 1−g=0.8) and $\theta_{max}=\text{2}\text{5}\text{0}$ for 20% of the period (i.e., g=0.2). In this scenario, we need to compute the selection gradients averaged over one period of the fluctuation. To find the values of $\alpha^{\cdot }$ and $\phi^{\cdot }$, we have to solve ${\hat{S}}_{\alpha }={\hat{S}}_{\phi }=\text{0}$ numerically. But in contrast to the constant scenario, we do not have ${\hat{S}}_{\alpha }\propto {\hat{S}}_{\phi }$ in a fluctuating environment and there is a single pair of values for $\alpha^{\cdot }$ and $\phi^{\cdot }$ (a coESS) that verify ${\hat{S}}_{\alpha }={\hat{S}}_{\phi }=\text{0}$ under the default parameter values of Table 1 (S2 Fig). More specifically, we find: $\alpha^{\cdot }=\text{0}.\text{0}\text{1}\text{9}\text{7}$ and $\phi^{\cdot }=\text{0}.\text{3}\text{2}\text{5}$.

**Without plasticity to arbitrium but with plasticity to stress:** Next, we allow some heterogeneity in the stress level among host cells (i.e., σ=0.1), under the same fluctuating environment. To find the values of the coESS $\alpha^{*\cdot }$ and $\phi^{*\cdot }$ of the virus we have to solve ${\hat{S}}_{\alpha^{*}}={\hat{S}}_{\phi^{*}}=\text{0}$ numerically, using the values $\alpha^{\cdot }=\text{0}.\text{0}\text{1}\text{9}\text{7}$ and $\phi^{\cdot }=\text{0}.\text{3}\text{2}\text{5}$ in normal cells (see above section) and the default parameter values of Table 1 (S3 Fig). More specifically, we find: $\alpha^{*\cdot }=\text{0}.\text{063}$ and $\phi^{*\cdot }=\text{0}$.

**With plasticity to arbitrium and without to stress:** In this scenario, we allow the lysis/lysogeny decision (i.e., the life-history strategies α and ϕ) to vary with time but without stress (i.e., σ=0). More specifically, we allow ϕ and α to be a function (i.e., a norm of reaction) of the time-varying concentration of arbitrium A(t). In principle, these functions could take different forms, but for the sake of simplicity we chose to use the following functions:


$$\alpha (t)=\alpha_{max}\left(\text{1}-H_{k}\left(\frac{A(t)}{A_{\alpha }}-\text{1}\right)\right)$$

$$\phi (t)=\phi_{max}H_{k}\left(\frac{A(t)}{A_{\phi }}-\text{1}\right)$$ (12)

Where we use a smooth approximation of the Heaviside step function:


$$H_{k}(x)=\frac{\text{1}}{\text{2}}+\frac{Arctan(kx)}{\pi }$$

Next, we want to determine the direction acting on the parameters that govern the shape of the plasticity function. When the coefficient of the transition matrix 𝐌 depend on a trait z only through its effect on X(t), we can use the chain rule in equation (4) to obtain:


$$S_{z}(t)=v(t).\frac{\partial M(t)}{\partial X(t)}.f(t)\frac{\partial X(t)}{\partial z}$$ (13)

Using equation (12) and (13), the gradients of selection on $A_{\alpha }$ and $A_{\phi }$ are:


$$S_{A_{\alpha }}(t)=v(t).\frac{\partial M(t)}{\partial \alpha (t)}.f(t)\frac{\partial \alpha (t)}{\partial A_{\alpha }}=-S_{\alpha }(t)\frac{\partial H_{k}\left(\frac{A(t)}{A_{\alpha }}-\text{1}\right)}{\partial A_{\alpha }}$$

$$S_{A_{\phi }}(t)=v(t).\frac{\partial M(t)}{\partial \phi (t)}.f(t)\frac{\partial \phi (t)}{\partial A_{\phi }}=S_{\phi }(t)\frac{\partial H_{k}\left(\frac{A(t)}{A_{\phi }}-\text{1}\right)}{\partial A_{\phi }}$$ (14)

which yields:


$${\hat{S}}_{A_{\alpha }}=\alpha_{max}\left\langle S_{\alpha }(t)\frac{kA(t)}{A_{\alpha }\pi \left({A_{\alpha }}^{\text{2}}+k^{\text{2}}{\left(A(t)-A_{\alpha }\right)}^{\text{2}}\right)}\right\rangle$$

$${\hat{S}}_{A_{\phi }}={-\phi }_{max}\left\langle S_{\phi }(t)\frac{kA(t)}{A_{\phi }\pi \left({A_{\phi }}^{\text{2}}+k^{\text{2}}{\left(A(t)-A_{\phi }\right)}^{\text{2}}\right)}\right\rangle$$ (15)

In the following, we focus on scenarios where we assume that k=10 (i.e., a smooth transition around at the threshold).

Similarly, we can compute the selection gradient on $\alpha_{max}$ and $\phi_{max}$ which yields:


$${\hat{S}}_{\alpha_{max}}=\left\langle S_{\alpha }(t)\left(\text{1}-H_{k}\left(\frac{A(t)}{A_{\alpha }}-\text{1}\right)\right)\right\rangle$$

$${\hat{S}}_{\phi_{max}}=\left\langle S_{\phi }(t)\left(H_{k}\left(\frac{A(t)}{A_{\phi }}-\text{1}\right)\right)\right\rangle$$ (16)

Again, to limit the number of free parameters in the model, we do not use equation (16) to study the coevolution of $\alpha_{max}$ and $\phi_{max}$ with $A_{\alpha }$ and $A_{\phi }$ but we assume that $\alpha_{max}$ and $\phi_{max}$ are fixed and equal to the ESS values when α and ϕ are not plastic (i.e., $\alpha_{max}=\alpha^{\cdot }=\text{0}.\text{0}\text{1}\text{9}\text{7}$ and $\phi_{max}=\phi^{\cdot }=\text{0}.\text{3}\text{2}\text{5}$). Next, we study numerically the coevolution between the traits $A_{\alpha }$ and $A_{\phi }$ under the parameter values of Table 1. S4 Fig shows the direction of evolution on these two traits. Using equation (15), we have to solve ${\hat{S}}_{A_{\alpha }}={\hat{S}}_{A_{\phi }}=\text{0}$ numerically. We find a single coESS strategy that verify this condition: $A_{\alpha }^{\cdot }=\text{0}.\text{0}\text{9}\text{7}$ and $A_{\phi }^{\cdot }=\text{0}.\text{0}\text{6}\text{3}$.

**With plasticity to arbitrium and plasticity to stress:** Finally, we consider the full model where the environment fluctuates periodically and the cells are exposed to some stress (i.e., σ=0.1). In normal (unstressed) cells the virus follows the strategy defined in equation (12). In stressed cells, however, the virus may adopt a different strategy:


$$\alpha^{*}(t)=\alpha_{max}^{*}\left(\text{1}-H_{k}\left(\frac{A(t)}{A_{\alpha^{*}}}-\text{1}\right)\right)$$

$$\phi^{*}(t)=\phi_{max}^{*}H_{k}\left(\frac{A(t)}{A_{\phi^{*}}}-\text{1}\right)$$ (17)

and the selection gradients on $A_{\phi }$ and $A_{\alpha }$ are (see equation (15)):


$${\hat{S}}_{A_{\alpha^{*}}}=\alpha_{max}^{*}\left\langle S_{\alpha }(t)\frac{kA(t)}{A_{\alpha^{*}}\pi \left({A_{\alpha^{*}}}^{\text{2}}+k^{\text{2}}{\left(A(t)-A_{\alpha^{*}}\right)}^{\text{2}}\right)}\right\rangle$$

$${\hat{S}}_{A_{\phi^{*}}}=-\phi_{max}^{*}\left\langle S_{\phi }(t)\frac{kA(t)}{A_{\phi^{*}}\pi \left({A_{\phi^{*}}}^{\text{2}}+k^{\text{2}}{\left(A(t)-A_{\phi^{*}}\right)}^{\text{2}}\right)}\right\rangle$$ (18)

Based on the previous scenarios, we fixed the maximum values of the functions for α, ϕ,$\alpha^{*}$ and $\phi^{*}$ ($\alpha_{max}=\text{0}.\text{0}\text{1}\text{9}\text{7}$, $\phi_{max}=\text{0}.\text{3}\text{2}\text{5}$, $\alpha_{max}^{*}=\text{0}.\text{0}\text{6}\text{3}$, $\phi_{max}^{*}=\text{0}$) and we used (13a) and (16) to solve ${\hat{S}}_{A_{\alpha }}={\hat{S}}_{A_{\phi }}={\hat{S}}_{A_{\alpha^{*}}}=\text{0}$ numerically (note that ${\hat{S}}_{A_{\phi^{*}}}=\text{0}$ is always verified because $\phi_{max}^{*}=\text{0})$. We find a single coESS strategy that verify this condition: $A_{\alpha }^{\cdot }=\text{0}.\text{0}\text{7}\text{8}$, $A_{\phi }^{\cdot }=\text{0}.\text{0}\text{6}\text{6}$, $A_{\alpha^{*}}^{\cdot }=\text{0}.\text{0}\text{9}\text{9}$. The simulation model used to obtain this coESS strategy will be be available from Zenodo after the acceptance of the manuscript.

### Evolutionary stable norms of reaction of the lysis/lysogeny decision of a temperate phage as a function of stress and arbitrium

The above scenario accounts for two realistic features of the environment: fluctuations of the availability of the resource (temporal variation in susceptible cells density) and heterogeneity in stress among host cells (variation among host cells). Our model allows us to demonstrate the adaptive value of plastic life-history strategies for the virus. More specifically, we predict how the virus should alter its lysis/lysogeny decision after (i) a variation in the concentration of arbitrium and (ii) a change in the level of stress of its host cell. Of course, these quantitative predictions are expected to depend on the parameter values that characterize the epidemiological scenario (e.g., the period and the amplitude of the fluctuations of the environment). Yet, we contend that these predictions are qualitatively robust and consistent with the experimental observations in SPbeta phage.

Fig 2A illustrates the epidemiological dynamics for the default parameters given in Table 1. These epidemiological dynamics drive the periodic fluctuations of the concentration of arbitrium (Fig 2B) and the periodic fluctuations of the selection for reactivation in normal and stressed cells given in equations (7a) and (7b), respectively (Fig 2C). Finally, Fig 2D, presents the norm of reactions predicted by our model (gradients of selection given by equations (15) and (18) under the default parameter values of Table 1.

## 7. Experiments

### Experimental model and subject details

*Bacillus subtilis* 168 and *Bacillus subtilis 168∆recA* were obtained from the *Bacillus* Genetic Stock Centre (BGSC). All phage used in this study are derivatives of the WT phi3T, also obtained from the BGSC. A signal-negative mutant *phi3T*∆*aimP::spc* and an arbitrium system mutant *phi3T∆aimRPX::spc*, where phi3T_5(yokI) has been replaced with a spectinomycin resistance cassette, were constructed as described previously [11]. The presence of the spectinomycin resistance cassette has no significant influence on virion production (S13 Fig). All infections were carried out in LB media. Strains were cultured in either 6 ml of LB media in a 30 ml glass universal vial or 1.5 ml of LB media in a 24-well plate at 37°C and shaking at 200 rpm. Antibiotics were used as follows unless otherwise stated: spectinomycin (100 mg/mL).

### Experimental methods

#### Host survival in response to stress.

To quantify host survival in response to stress, we inoculated individual colonies of *B. subtilis* 168 and *B. subtilis* 168*∆recA* into 6 ml of LB media and incubated shaking overnight at 37°C and 200 rpm. We washed overnight cultures 3× in 6 ml of 1×M9 salts before resuspending cells in 6 mL of fresh LB media. We diluted the washed cultures to ~4 × 10^5^ cells/mL in 1.5 mL of LB media in a 24-well plate with either 0, 50, or 100 ng/mL of MMC and incubated for 8 hours at 37°C and 200 rpm. After 8 hours, we serially diluted cultures in 1×M9 and plated onto LB agar. Plates were incubated overnight at 37°C and the number of CFU/mL calculated.

#### Prophage reactivation and host survival in response to stress.

To quantify prophage reactivation and host survival under stressful conditions, we inoculated individual *B. subtilis* 168*::phi3T∆aimP* or *B. subtilis* 168∆recA::phi3T∆aimP lysogens into 6 ml of LB media containing 100 ng/mL of spectinomycin and incubated shaking overnight at 37°C and 200 rpm. We diluted the washed cultures to ~4 × 10^5^ cells/mL in 1.5 mL of LB media in a 24-well plate with either 0, 50, or 100 ng/mL of MMC and incubated for 8 hours at 37°C and 200 rpm. After 8 hours, we serially diluted cultures in 1×M9 and plated onto LB agar containing 100 ng/mL of spectinomycin to quantify lysogen survival. Plates were incubated overnight at 37°C and the number of CFU/mL calculated. To quantify virion production, we sampled 100 µl of culture into chloroform to lyse remaining bacterial cells before centrifuging samples for 10 min at 3,500*g* to remove cellular debris. Supernatant containing phage from prophage reactivation was then used in small-drop plaque assays to calculate sample PFU/mL. Log-phase cultures of *B. subtilis* 168 were mixed with LB media supplemented with 0.1 mM MnCl_2_, 5 mM MgCl_2_, and 0.75% agar, and added to LB agar plates containing 0.1 mM MnCl_2_ and 5 mM MgCl_2_. Phage-containing supernatant was serially diluted and 10 µl of each dilution spotted onto bacterial lawns. Plates were incubated overnight at 37°C and the number of PFU/mL calculated. *B. subtilis* 168 contains the SPbeta prophage. To rule out SPbeta forming plaques and biasing our results, we performed plaque assays which demonstrate that phi3T and mutant phage plaque on *B. subtilis* 168, but SPbeta does not (S12 Fig).

#### Independence of response to signal and stress.

To quantify prophage reactivation response to signal in the absence of stress, we inoculated individual *B. subtilis* 168*∆recA::phi3T∆aimP* lysogens into 6 ml of LB media containing 100 ng/mL of spectinomycin and incubated shaking overnight at 37°C and 200 rpm. We washed overnight cultures of lysogens 3× in 6 ml of 1×M9 salts before resuspending cells in 6 mL of fresh LB media containing either 0 nM or 1,000 nM of signaling peptide. We then diluted to ~4 × 10^5^ cells/mL in 1.5 mL of LB media in a 24-well plate containing either 0 nM signaling peptide or 1,000 nM of signaling peptide and plates were incubated for 8 hours at 37°C and 200 rpm. To quantify virion production after 8 hours, we sampled 100 µl of culture into chloroform to lyse remaining bacterial cells before centrifuging samples for 10 min at 3,500*g* to remove cellular debris. Supernatant containing phage from prophage reactivation was then used in small-drop plaque assays to calculate sample PFU/mL. Log-phase cultures of *B. subtilis* 168 were mixed with LB media supplemented with 0.1 mM MnCl_2_, 5 mM MgCl_2_, and 0.75% agar, and added to LB agar plates containing 0.1 mM MnCl_2_ and 5 mM MgCl_2_. Phage-containing supernatant was serially diluted and 10 µl of each dilution spotted onto bacterial lawns. Plates were incubated overnight at 37°C and the number of PFU/mL calculated.

To quantify prophage reactivation response to stress in the absence of signaling, we inoculated individual *B. subtilis* 168*::phi3T∆aimRPX* lysogens into 6 ml of LB media containing 100 ng/mL of spectinomycin and incubated shaking overnight at 37°C and 200 rpm. We diluted the washed cultures to ~4 × 10^5^ cells/mL in 1.5 mL of LB media in a 24-well plate with either 0 or 50 ng/mL of MMC and incubated for 8 hours at 37°C and 200 rpm. Virion production after 8 hours was quantified as above.

#### Prophage reactivation, signal, and stress.

To quantify prophage reactivation and host survival in response to signal and stress, we inoculated individual *B. subtilis* 168*::phi3T∆aimP* lysogens into 6 ml of LB media containing 100 ng/mL of spectinomycin and incubated shaking overnight at 37°C and 200 rpm. We washed overnight cultures of lysogens 3× in 6 ml of 1×M9 salts before resuspending cells in 6 mL of fresh LB media containing either 0, 10, 100, or 1,000 nM of signaling peptide. We then diluted the washed cultures to ~4 × 10^5^ cells/mL in 1.5 mL of LB media with the appropriate concentration of signaling peptide in a 24-well plate and either 0, 50, or 100 ng/mL of MMC before plates were incubated for 8 hours at 37°C and 200 rpm.

After 8 hours, we serially diluted cultures in 1×M9 and plated onto LB agar containing 100 ng/mL of spectinomycin to quantify lysogen survival. Plates were incubated overnight at 37°C and the number of CFU/mL calculated. To quantify virion production, we sampled 100 µl of culture into chloroform to lyse remaining bacterial cells before centrifuging samples for 10 min at 3,500*g* to remove cellular debris. Supernatant containing phage from prophage reactivation was then used in small-drop plaque assays to calculate sample PFU/mL. Log-phase cultures of *B. subtilis* 168 were mixed with LB media supplemented with 0.1 mM MnCl_2_, 5 mM MgCl_2_, and 0.75% agar, and added to LB agar plates containing 0.1 mM MnCl_2_ and 5 mM MgCl_2_. Phage-containing supernatant was serially diluted and 10 µl of each dilution spotted onto bacterial lawns. Plates were incubated overnight at 37°C and the number of PFU/mL calculated.

#### Lysogen formation, signal, and stress.

To quantify lysogen formation in the presence or absence of stress (MMC) and signaling peptide we inoculated individual colonies of *B. subtilis* 168 into 6 ml of LB media and incubated shaking overnight at 37°C and 200 rpm. We washed overnight cultures of lysogens 3× in 6 ml of 1×M9 salts before resuspending cells in 6 mL of fresh LB media containing either 0 nM signaling peptide or 1,000 nM of signaling peptide. Cultures were incubated at 37°C shaking for a further 2 hours. Exponentially growing cultures were then diluted to an OD of 0.2 in 1.5 mL of LB media in a 24-well plate containing either 0 nM signaling peptide or 1,000 nM of signaling peptide and either 0, 50, or 100 ng/mL of MMC was added to each replicate well. Cultures were incubated for 20 m at 37°C shaking before phage phi3T∆aimP:spc was added to each replicate well at an MOI of 0.1 and a single round of infection was allowed to take place (40 m). To quantify total CFU, cultures were serially diluted in 1×M9 and plated onto LB agar. Plates were incubated overnight at 37°C and the number of CFU/mL calculated. To quantify lysogen formation, cultures were serially diluted in 1×M9 and plated onto LB agar containing 100 ng/µl of spectinomycin. To quantify virion production, we sampled 100 µl of culture into chloroform to lyse bacterial cells before centrifuging samples for 10 min at 3,500*g* to remove cellular debris. Supernatant containing phage from lytic infections was then used in small-drop plaque assays to calculate sample PFU/mL. Log-phase cultures of *B. subtilis* 168 were mixed with LB media supplemented with 0.1 mM MnCl_2_, 5 mM MgCl_2_, and 0.75% agar, and added to LB agar plates containing 0.1 mM MnCl_2_ and 5 mM MgCl_2_. Phage-containing supernatant was serially diluted and 10 µl of each dilution spotted onto bacterial lawns. Plates were incubated overnight at 37°C and the number of PFU/mL calculated.

#### Detection of recombination events.

*B. subtilis* 168 was grown up overnight in LB (37°C, 200 rpm) from a single colony, diluted 1:100 and allowed to grow to 0.6 OD_600_. 500 µL of 0.6 OD_600_ culture was added to 25 mL of fresh LB (Mn/Mg) and four treatments were applied in three biological replicates:

1. *B. subtilis* 168 only (−ve control) ×3
2. *B. subtilis* 168 + MMC 50 ng/mL (control) ×3
3. *B. subtilis* 168 + phi3T (MOI ~ 0.1) ×3
4. *B. subtilis* 168 + phi3T (MOI ~ 0.1) + MMC 50 ng/mL×x3

Samples were left overnight at 37°C, shaking at 200 rpm, before centrifugation at 10,000*g* at 4°C for 10 min and syringe filtered through a 0.22 µm pore filter. Polyethylene glycol 8000 (PEG) and NaCl were dissolved in the lysate to final concentrations of 10% w/v and 1.12 M, respectively, and left overnight at 4°C. Phage were concentrated by centrifugation at 10,000*g* for 10 min, the supernatant removed and the phage pellet re-suspended in 750 mL of SM buffer (100 mM NaCl, 50 mM Tris-HCL, 8 mM MgSO_4_-H_2_O). Phage lysates were treated with DNase 1 (5 mg/mL, Roche) and RNase A (10 mg/mL, Invitrogen) for 30 min at 37°C to remove non-encapsulated (e.g., free bacterial DNA) nucleic acids from the lysate, enzyme activity was terminated with heat treatment. DNA was purified using DNeasy blood and tissue kit following the manufacturer’s instructions and quantified using a broad-range Qubit dsDNA Quantification Assay kit and normalized to 200 ng in 30 µL. As expected, no DNA was detected in the control lysate because of low Spbeta induction, so these samples were omitted from sequencing. Genomic DNA was prepared for sequencing by the Exeter Sequencing Service using the NEBNext Ultra II FS DNA PCR-Free Library Prep Kit (New England Biolabs), following the manufacturer’s protocol. Libraries were dual-indexed using unique barcodes and pooled for multiplexing. Sequencing was performed on the Illumina NovaSeq platform using paired-end 150 bp reads that were filtered and trimmed using MultiQC (v. 10.1) [44].

Whole genome comparison of Spbeta (134,416 bp; NCBI accession: NC_001884.1) and phi3T (128,375 bp; NCBI accession: KY030782.1) was performed with the progressiveMauve algorithm [45] in Geneious v. 10.1 (https://www.geneious.com) revealing high genetic similarity between phage (47.7% pairwise nucleotide identity). To facilitate detection of SPbeta and phi3T replication in mixed phage lysates, and recombination between the phage, reference genomes were generated per phage containing the concatenated fragments unique to SPbeta and phi3T genomes. The two genomes were first re-aligned using nucmer (MUMmer4 v4.0; [46]) with permissive parameters to ensure that identified unique regions were genuinely unique. Aligned regions were defined using delta-filter with a minimum identity threshold of 85% and a minimum alignment length of 100 bp (-i 85 -l 100), allowing for the identification of all moderately conserved sequences between the genomes. Aligned regions were then subtracted from the original reference sequences using BEDTools v2.31.1 [47]. To confirm that the remaining regions were unique to each phage, each ‘unique’ FASTA file was used as a query in a BLASTn v. 2.15.0 search (e-value threshold 1e^−5^) [48] against a database constructed from the opposing phage genome. Fragments with significant hits were removed from the unique region files using BEDTools. The remaining fragments were then concatenated into single unique reference FASTA sequences for phi3T and SPbeta*.*

For evidence of phi3T and MMC-induced activation of SPbeta in *Bacillus subtilis* 168, reads from one sample per treatment (phi3T-only, MMC, and the combination) were aligned to the SPbeta unique reference using BWA-MEM with the -M flag [49], and converted to sorted and indexed BAM files using SAMtools [50]. Resulting alignments were visualized in Geneious v10.1. Four regions (19–31 bp in length) showed markedly elevated coverage in both the phi3T-only and phi3T + MMC treatment samples. These regions were determined to be non-unique to SPbeta and were manually removed in Geneious v.10.1. Coverage data were exported from Geneious as a CSV file and visualized using ggplot2 in R [51]. To determine replication of phi3T during infection of *B. subtilis* 168, the methods above were repeated against the phi3T unique reference.

To detect recombination events, the unique reference sequences for phi3T and *SPbeta* were combined, and reads from three biological replicates per treatment were mapped to the merged reference using the approach described above. Discordant read pairs—defined as paired-end reads where one mate mapped to phi3T and the other to *SPbeta*—were extracted from the sorted, indexed BAM files. No discordant reads were detected in these samples. To evaluate the sensitivity of the method, control DNA mixtures of phi3T WT and a chimeric phi3T carrying a heterologous aimR gene (phi3T*.SIIIRGA*; IMG gene ID: 2717055481) were prepared at 1:10 and 1:1,000 chimera:WT ratios and sequenced. Reads were mapped to a combined reference containing the phi3T WT genome (KY030782.1) and the chimeric aimR gene sequence. Discordant read pairs were extracted and visualized using Geneious v10.1.

#### Quantification and statistical analysis.

Statistical analyses were conducted with an alpha level of 0.05 using R Core Team [51]. Data were analyzed using linear models and multiple linear regression. All figures of experimental data are presented on the log_10_ scale. Non-significant factors and interactions between factors were removed sequentially using backward elimination. The significance level of each factor was determined by comparing the final model with and without the factor of interest using *F*-tests. Diagnostic plots (residuals versus fitted, *Q*–*Q*, residuals versus leverage, and scale-location plots) were used to check model fit and that the assumptions of linear regression were not violated. For statistical analysis of lysogen with stress (MMC) and signal (SAIRGA) treatments (Fig 4B), log_10_-transformed CFU/mL values were used due to the data spanning several orders of magnitude under MMC treatment. This transformation stabilized variance and ensured linear model assumptions (homoscedasticity and normality of residuals) were met. Similarly, for virion production when tested with stress and signaling peptide (Fig 5B), log_10_ transformation of PFU/mL values were applied to stabilize variance and improve normality, as confirmed by diagnostic plots and residual simulation. For all other datasets, analyses were performed on untransformed values, as variance remained approximately constant across treatments. Pairwise comparisons were performed on estimate marginal means from the fitted models, using the emmeans package [52], with *p*-values adjusted for multiple comparisons using the Tukey method. These adjusted p-values are reported throughout the results section.

## Supporting information

S1 Fig Evolution of fixed rates of lysogenisation and reactivation in a constant environment with no stress. (DOCX)

S2 Fig Evolution of fixed rates of lysogenisation and reactivation in a fluctuating environment with no stress. (DOCX)

S3 Fig Evolution of rates of lysogenisation and reactivation in a fluctuating environment with stress. (DOCX)

S4 Fig Evolution of the response of lysogenisation and reactivation to arbitrium concentration in a fluctuating environment without stress. (DOCX)

S5 FigEffect of parameter values (adsorbtion rate a and burst size B) on epidemiological fluctuations and selection gradient.(DOCX)

S6 FigEffect of parameter values (death rates d and $d_{V}$) on epidemiological fluctuations and selection gradient.(DOCX)

S7 FigEffect of parameter values (recovery rate γ from stress) on epidemiological fluctuations and selection gradient.(DOCX)

S8 Fig Host background and rates of prophage excision. Prophage excision of Phi3T WT, Phi3TΔaimP, and L3 from *B. subtilis 168* WT and *B. subtilis 168Δ6* hosts.
(DOCX)

S9 Fig Phi3T and mitomycin C induce Spbeta from B. subtilis 168. Read depth of Spbeta in response to a) Phi3T infection, b) Mitomycin C, and c) both Phi3T and Mitomycin C.
(DOCX)

S10 Fig Phi3T is an active infection of *B. subtilis* 168. Coverage of reads mapped against unique Phi3T genome fragments.
(DOCX)

S11 Fig No recombination was detected between Spbeta and Phi3T in the treatment groups, but discordant reads were detected in the positive control groups. (DOCX)

S12 Fig Spbeta does not plaque on *B. subtilis* 168. Phi3TΔaimP, Phi3TΔaimP.spec, and SpBeta were spotted on top agar lawns containing exponential phase *B. subtilis* 168::Δ6 and *B. subtilis* 168 in 1:10 dilution series.
(DOCX)

S13 Fig There is no difference in rates of prophage excision from *B. subtilis* 168 between WT Phi3T and the mutant phi3TΔaimP with or without the spectinomycin cassette. (DOCX)

S1 Data Colony-forming units and plaque-forming units of *Bacillus subtillis 168* hosts with and without phi3T prophage exposed to both host stress (mitomycin C: MMC) and arbitrium signaling (SAIRGA signal peptide). **Data used to produce** Fig 3A–3F.
(XLSX)

S2 Data Colony-forming units and plaque-forming units of phi3T prophage exposed to combinations of host stress (mitomycin C: MMC) and arbitrium signaling (SAIRGA signal peptide). **Data used to produce** Fig 4A and 4B.
(XLSX)

S3 Data Colony-forming units and plaque-forming units of infections of *Bacillus subtillis 168* by phi3T∆*aimP*::spc exposed to combinations of host stress (mitomycin C: MMC) and arbitrium signaling (SAIRGA signal peptide). **Data used to produce** Fig 5A and 5B.
(XLSX)

S4 Data Plaque-forming units from prophage excision in different host backgrounds. **Data used to produce** Figs S8 and S13.
(XLSX)

## Abbreviations

BGSC: *Bacillus* Genetic Stock Centre
CFU: colony-forming units
MGEs: mobile genetic elements
MMC: mitomycin C
PFU: plaque-forming units
WT: wild-type
