# Supplementary material for: Temperate phage evolve to integrate host stress and quorum signals in lysis–lysogeny decisions
Source: PLoS Biol. 2026 Jan 5;24(1):e3003567. doi: 10.1371/journal.pbio.3003567 (PMC12768286; doi:10.1371/journal.pbio.3003567)
Supplement: S1 Fig — (DOCX) [file pbio.3003567.s001.docx]

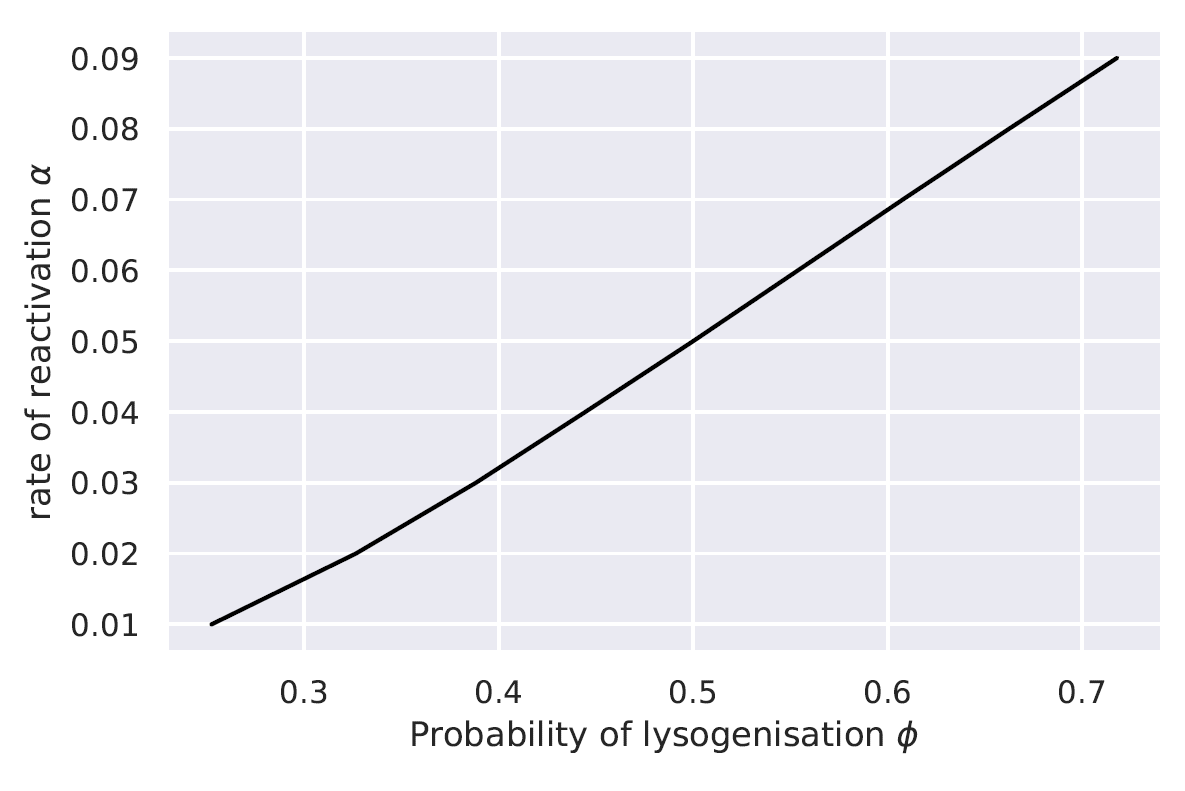


**Figure S1. Evolution of fixed rates of lysogenisation and reactivation in a constant environment with no stress.** We use equations (7a) and (8a) to compute the selection gradient on $\alpha$ and $\phi$ when $\left( t \right)=50$ . The blue arrows indicate selection on $\phi$, and the red arrows indicate selection on $\alpha$. All the points sitting on the black line verify ${\hat{\mathcal{S}}}_{\alpha}={\hat{\mathcal{S}}}_{\phi}=0.$ See Table 1 for other parameter values.
