# Supplementary material for: Temperate phage evolve to integrate host stress and quorum signals in lysis–lysogeny decisions
Source: PLoS Biol. 2026 Jan 5;24(1):e3003567. doi: 10.1371/journal.pbio.3003567 (PMC12768286; doi:10.1371/journal.pbio.3003567)
Supplement: S2 Fig — (DOCX) [file pbio.3003567.s002.docx]

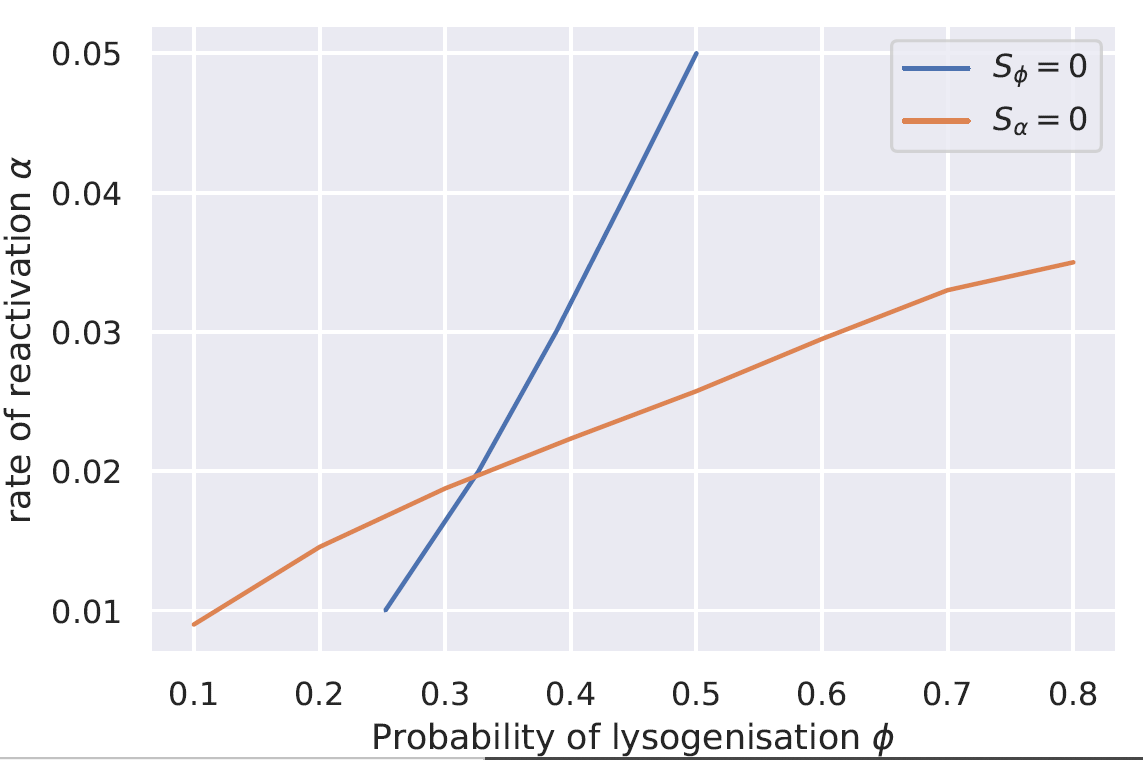


**Figure S2. Evolution of fixed rates of lysogenisation and reactivation in a fluctuating environment with no stress.** We use equations (7a) and (8a) to compute the selection gradient on $\alpha$ and $\phi$ in a periodic environment where $\theta\left( t \right)=\theta_{max}\boldsymbol{1}_{\left[ t/T< g \right]}$ with $\theta_{max}=250$ and $g=0.2$. The blue and orange line indicate trait values where ${\hat{\mathcal{S}}}_{\phi}=0$ and ${\hat{\mathcal{S}}}_{\alpha}=0$, respectively. The dot indicates the position of the evolutionary stable strategy (${\hat{\mathcal{S}}}_{\alpha}={\hat{\mathcal{S}}}_{\phi}=0)$: $\phi^{\bullet}=0.3$2 and $\alpha^{\bullet}=0.0197$. See Table 1 for other parameter values.
