# Supplementary material for: Temperate phage evolve to integrate host stress and quorum signals in lysis–lysogeny decisions
Source: PLoS Biol. 2026 Jan 5;24(1):e3003567. doi: 10.1371/journal.pbio.3003567 (PMC12768286; doi:10.1371/journal.pbio.3003567)
Supplement: S3 Fig — (DOCX) [file pbio.3003567.s003.docx]

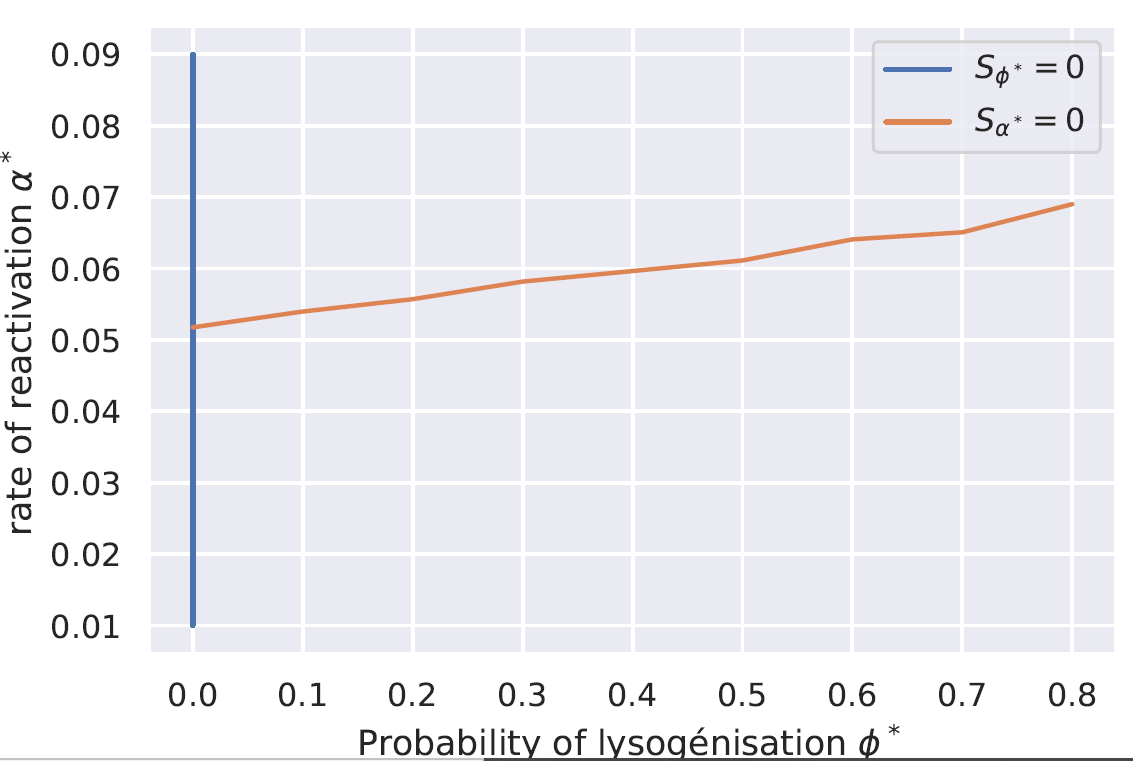


**Figure S3. Evolution of rates of lysogenisation and reactivation in a fluctuating environment with stress.** We use equations (7b) and (8b) to compute the selection gradient on $\alpha^{*}$ and $\phi^{*}$ in a periodic environment where $\theta\left( t \right)=\theta_{max}\boldsymbol{1}_{\left[ t/T< g \right]}$ with $\theta_{max}=250$ and $g=0.2$. We assume that the virus adopts the strategy $\phi^{\bullet}=0.3$2 and $\alpha^{\bullet}=0.0197$ in normal cells. The blue and orange line indicate trait values where ${\hat{\mathcal{S}}}_{\phi^{*}}=0$ and ${\hat{\mathcal{S}}}_{\alpha^{*}}=0$, respectively. The dot indicates the position of the evolutionary stable strategy (${\hat{\mathcal{S}}}_{\alpha^{*}}={\hat{\mathcal{S}}}_{\phi^{*}}=0)$: $\phi^{\bullet*}=0$ and $\alpha^{\bullet*}=0.052$. See Table 1 for other parameter values.
