# Supplementary material for: Temperate phage evolve to integrate host stress and quorum signals in lysis–lysogeny decisions
Source: PLoS Biol. 2026 Jan 5;24(1):e3003567. doi: 10.1371/journal.pbio.3003567 (PMC12768286; doi:10.1371/journal.pbio.3003567)
Supplement: S4 Fig — (DOCX) [file pbio.3003567.s004.docx]

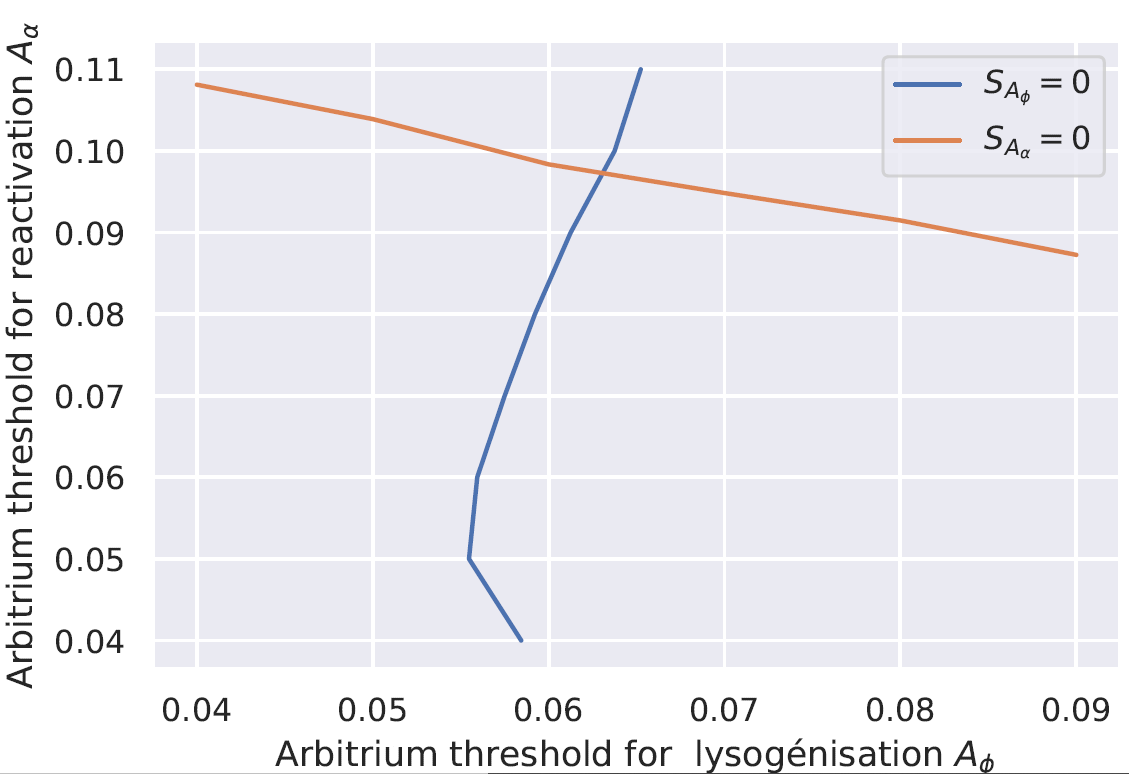


**Figure S4. Evolution of the response of lysogenisation and reactivation to arbitrium concentration in a fluctuating environment without stress.** We use equations (13a) to compute the selection gradient on $A_{\alpha}$ and $A_{\phi}$ in a periodic environment where $\theta\left( t \right)=\theta_{max}\boldsymbol{1}_{\left[ t/T< g \right]}$ with $\theta_{max}=250$ and $g=0.2$. The blue and orange line indicate trait values where ${\hat{\mathcal{S}}}_{A_{\phi}}=0$ and ${\hat{\mathcal{S}}}_{A_{\alpha}}=0$, respectively. The dot indicates the position of the evolutionary stable strategy (${\hat{\mathcal{S}}}_{A_{\phi}}={\hat{\mathcal{S}}}_{A_{\alpha}}=0)$: $A_{\phi}^{\bullet}=0.063$ and $A_{\alpha}^{\bullet}=0.097$. See Table 1 for other parameter values.
