# Supplementary material for: Temperate phage evolve to integrate host stress and quorum signals in lysis–lysogeny decisions
Source: PLoS Biol. 2026 Jan 5;24(1):e3003567. doi: 10.1371/journal.pbio.3003567 (PMC12768286; doi:10.1371/journal.pbio.3003567)
Supplement: S7 Fig — (DOCX) [file pbio.3003567.s007.docx]

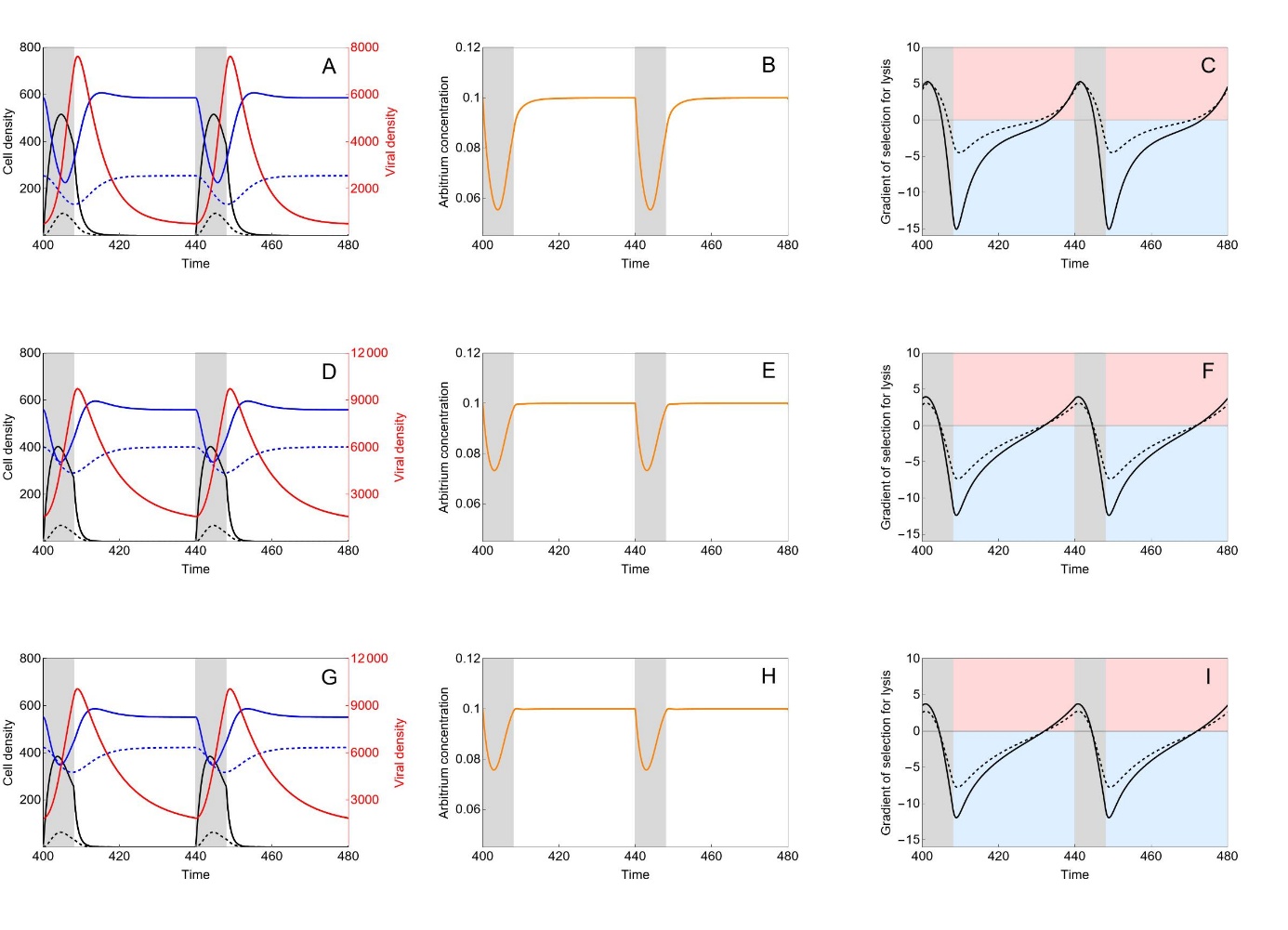


**Figure S7 : Effect of parameter values (recovery rate** $\gamma$ **from stress) on epidemiological fluctuations and selection gradient.**

We vary the recovery rate from stressed state: panels (A-C) $\gamma=0.1$, panels (D-F) $\gamma={10}^{-2}$, panels (G-I) $\gamma={10}^{-3}$. As in **Figure 2** we show the fluctuations of the density of the free virus (red) and different cell types (black: susceptible cells, blue: lysogen) where the full lines refer to non-stressed cells and dashed lines refer to stressed cells in panels (A, D, G). The gray shading indicates the time when there is an influx of new uninfected cells in the host population. We also show the fluctuations in Arbitrium signal concentration (orange) in panels (B, E, H). Finally, we show fluctuations of the sign of the gradient of selection for lysis in normal (full line: $Bv_{V}\left( t \right)-v_{L}\left( t \right)$) and stressed cells (dashed black line: $Bv_{V}\left( t \right)-v_{L^{*}}\left( t \right)$) in panels (C, F, I). The red shading indicates that lysis is favoured ($Bv_{V}\left( t \right)>v_{L}\left( t \right))$, while the blue shading indicates lysogeny is favoured ($Bv_{V}\left( t \right)<v_{L}\left( t \right))$. Crucially, the covariance between the selection for lysis and the concentration of Arbitrium is always negative for the three sets of parameters. This negative covariance is driving the plasticity of the virus in traits $\alpha$ and $\phi$ because low Arbitrium concentration coincides with a relatively high density of susceptible cells. See Table 1 for other parameter values.
