# Supplementary material for: Temperate phage evolve to integrate host stress and quorum signals in lysis–lysogeny decisions
Source: PLoS Biol. 2026 Jan 5;24(1):e3003567. doi: 10.1371/journal.pbio.3003567 (PMC12768286; doi:10.1371/journal.pbio.3003567)
Supplement: S8 Fig — Prophage excision of Phi3T WT, Phi3TΔaimP, and L3 from B. subtilis 168 WT and B. subtilis 168Δ6 hosts. (DOCX) [file pbio.3003567.s008.docx]

**
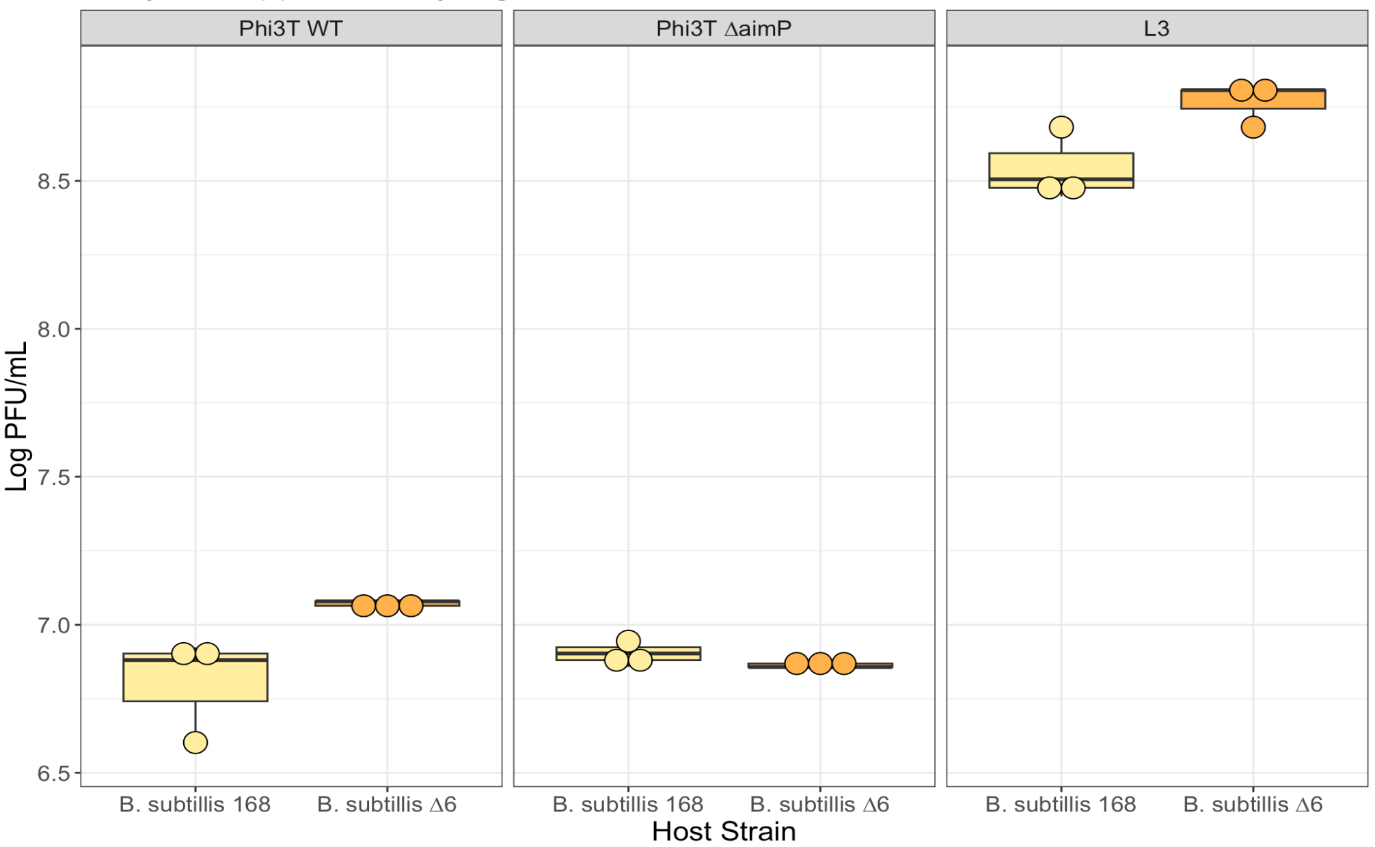
Figure S8. Host background and rates of prophage excision. Prophage excision of Phi3T WT, Phi3TΔaimP and L3 from *B. subtilis 168* WT and *B. subtilis 168Δ6* hosts. The data underlying figure S8 is available in S4 Data.**
