# Supplementary material for: Temperate phage evolve to integrate host stress and quorum signals in lysis–lysogeny decisions
Source: PLoS Biol. 2026 Jan 5;24(1):e3003567. doi: 10.1371/journal.pbio.3003567 (PMC12768286; doi:10.1371/journal.pbio.3003567)
Supplement: S9 Fig — Read depth of Spbeta in response to a) Phi3T infection, b) Mitomycin C, and c) both Phi3T and Mitomycin C. (DOCX) [file pbio.3003567.s009.docx]

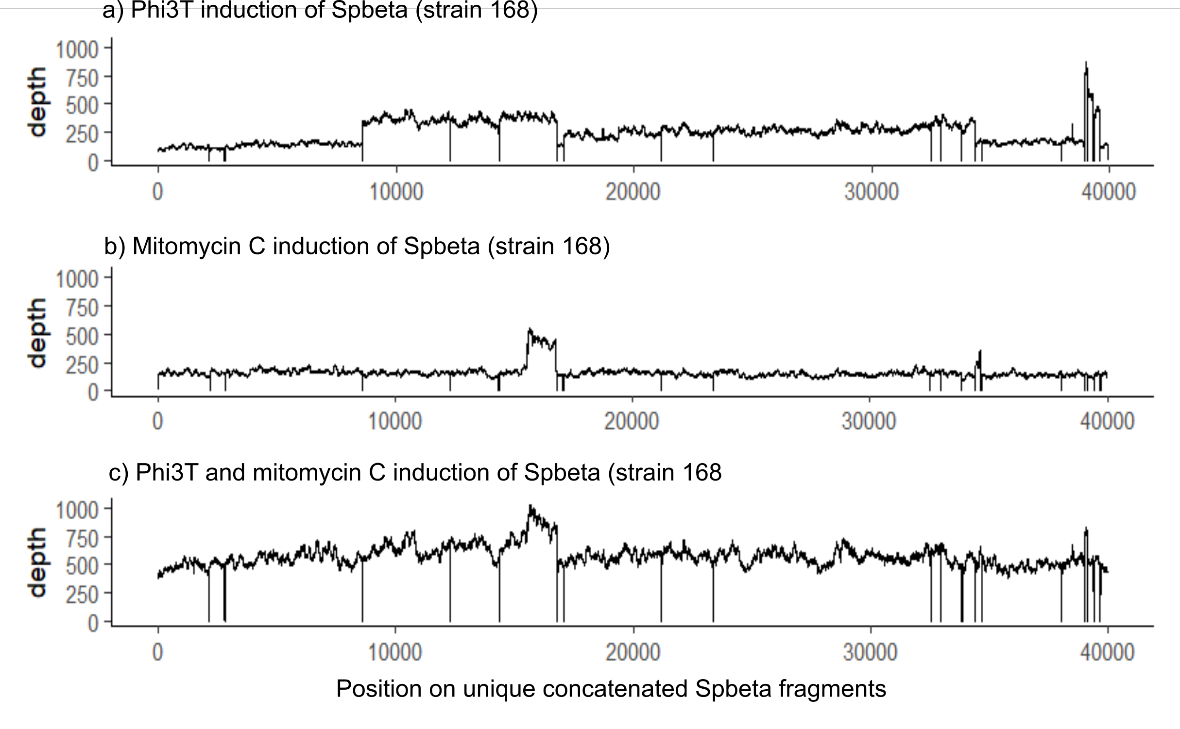


**Figure S9. Phi3T and mitomycin C induce Spbeta from B. subtilis 168. Read depth of Spbeta in response to a) Phi3T infection, b) Mitomycin C and c) both Phi3T and Mitomycin C. Sequencing data can be accessed through the NCBI BioProject PRJNA1365494.**
