# Supplementary material for: Temperate phage evolve to integrate host stress and quorum signals in lysis–lysogeny decisions
Source: PLoS Biol. 2026 Jan 5;24(1):e3003567. doi: 10.1371/journal.pbio.3003567 (PMC12768286; doi:10.1371/journal.pbio.3003567)
Supplement: S10 Fig — Coverage of reads mapped against unique Phi3T genome fragments. (DOCX) [file pbio.3003567.s010.docx]

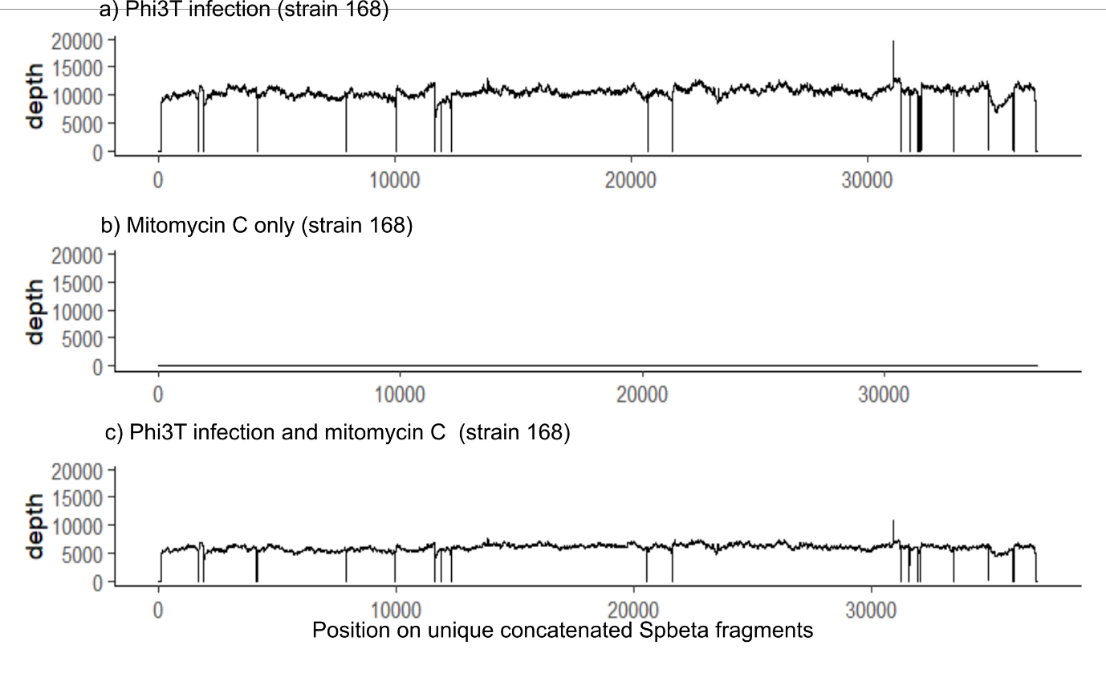
**Figure S10. Phi3T is an active infection of *B. subtilis* 168. Coverage of reads mapped against unique Phi3T genome fragments. Sequencing data can be accessed through the NCBI BioProject PRJNA1365494.**
