# Supplementary material for: Temperate phage evolve to integrate host stress and quorum signals in lysis–lysogeny decisions
Source: PLoS Biol. 2026 Jan 5;24(1):e3003567. doi: 10.1371/journal.pbio.3003567 (PMC12768286; doi:10.1371/journal.pbio.3003567)
Supplement: S11 Fig — (DOCX) [file pbio.3003567.s011.docx]

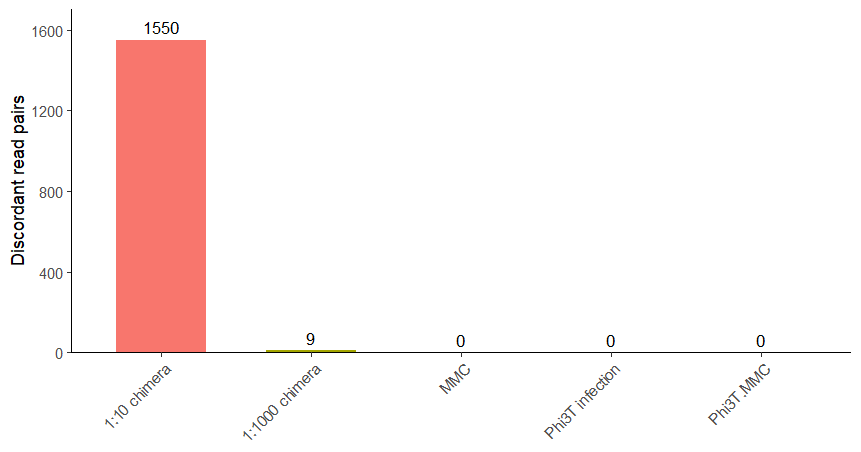


**Figure S11. No recombination was detected between Spbeta and Phi3T in the treatment groups, but discordant reads were detected in the positive control groups. Sequencing data can be accessed through the NCBI BioProject PRJNA1365494.**
