# Supplementary material for: Temperate phage evolve to integrate host stress and quorum signals in lysis–lysogeny decisions
Source: PLoS Biol. 2026 Jan 5;24(1):e3003567. doi: 10.1371/journal.pbio.3003567 (PMC12768286; doi:10.1371/journal.pbio.3003567)
Supplement: S12 Fig — Phi3TΔaimP, Phi3TΔaimP.spec, and SpBeta were spotted on top agar lawns containing exponential phase B. subtilis 168::Δ6 and B. subtilis 168 in 1:10 dilution series. (DOCX) [file pbio.3003567.s012.docx]

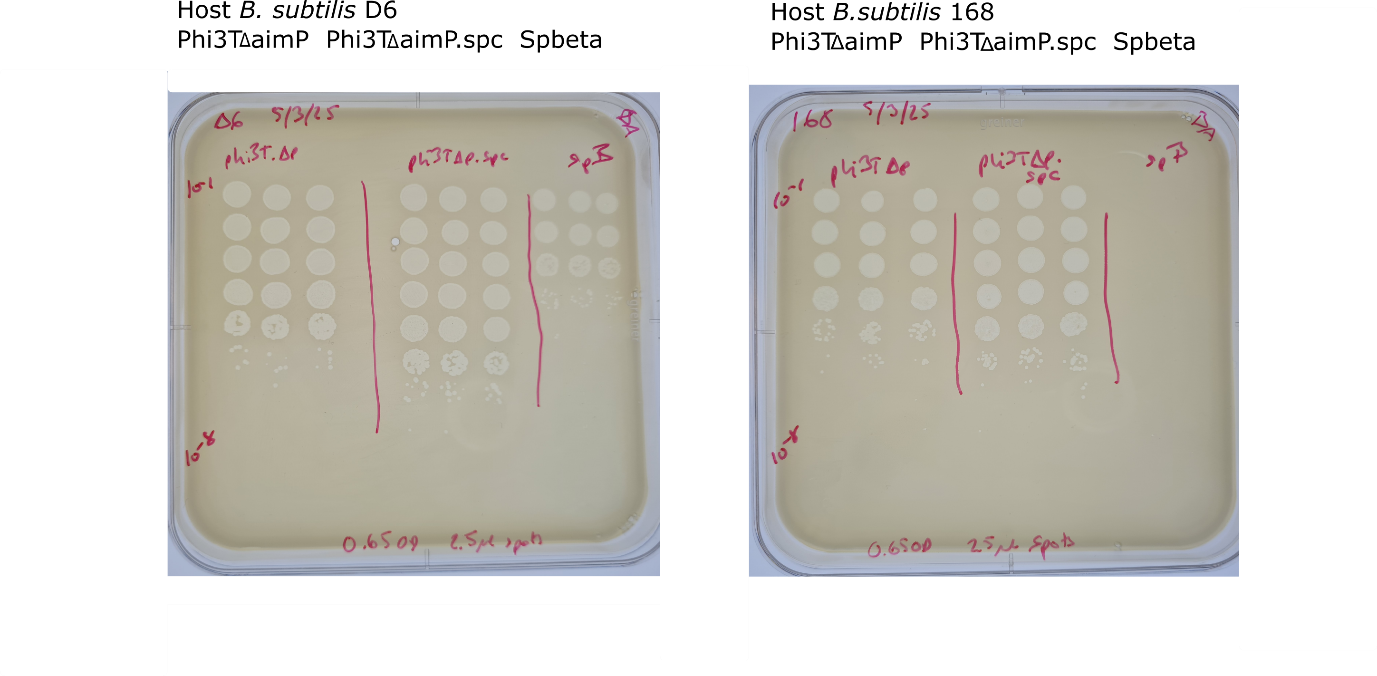


**Fig.S12 Spbeta does not plaque on *B. subtilis* 168. Phi3TΔaimP, Phi3TΔaimP.spec and SpBeta were spotted on top agar lawns containing exponential phase *B. subtilis* 168::Δ6 and *B. subtilis* 168 in 1:10 dilution series.**
