# Supplementary material for: Temperate phage evolve to integrate host stress and quorum signals in lysis–lysogeny decisions
Source: PLoS Biol. 2026 Jan 5;24(1):e3003567. doi: 10.1371/journal.pbio.3003567 (PMC12768286; doi:10.1371/journal.pbio.3003567)
Supplement: S13 Fig — (DOCX) [file pbio.3003567.s013.docx]

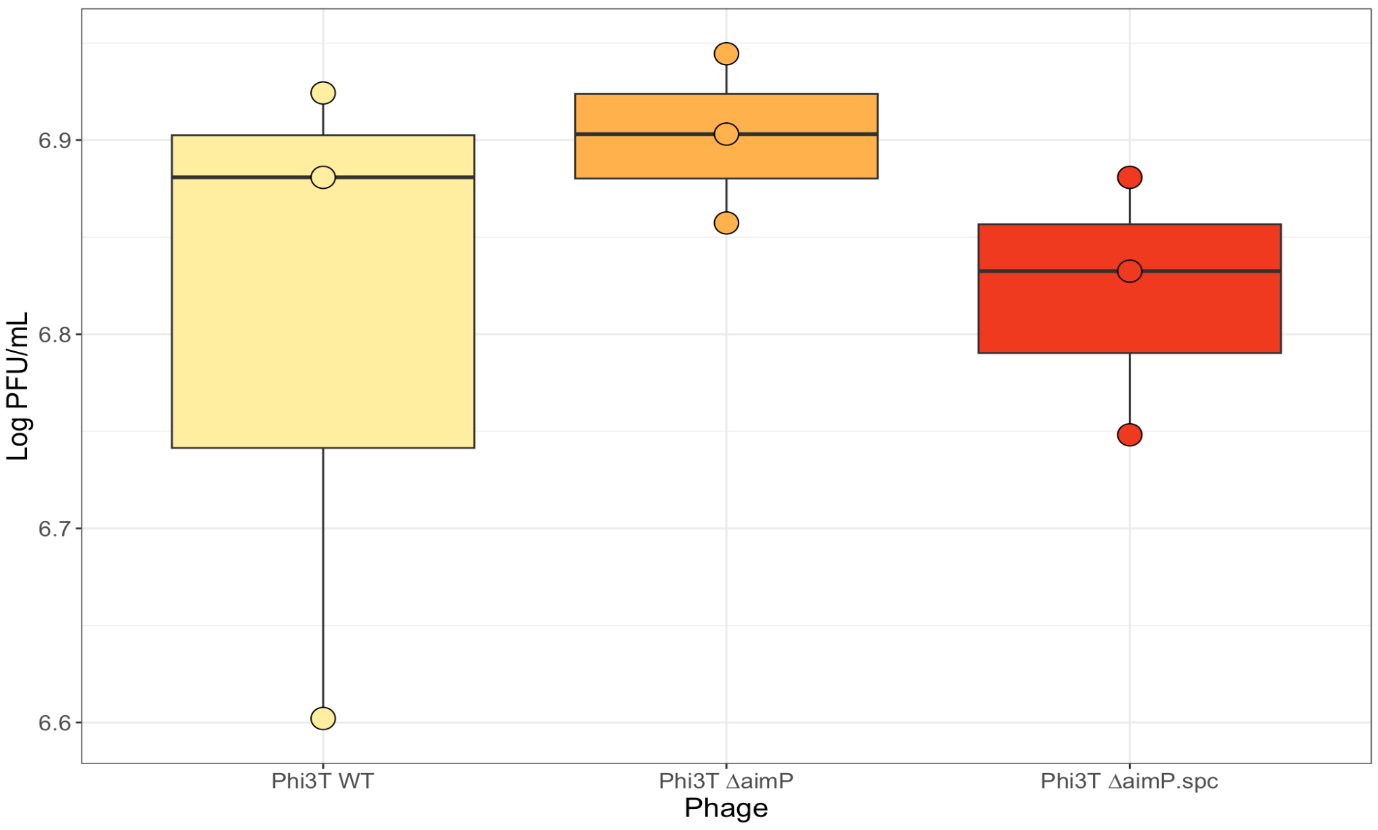


**Figure S13. There is no difference in rates of prophage excision from *B. subtilis* 168 between WT Phi3T and the mutant phi3TΔaimP with or without the spectinomycin cassette. The data underlying figure S13 is available in S4 Data.**
